# Humin Formation on SBA-15-pr-SO_3_H Catalysts during the Alcoholysis of Furfuryl Alcohol to Ethyl Levulinate: Effect of Pore Size on Catalyst Stability, Transport, and Adsorption

**DOI:** 10.1021/acsami.3c04613

**Published:** 2023-05-15

**Authors:** Graziano Di Carmine, Costanza Leonardi, Luke Forster, Min Hu, Daniel Lee, Christopher M. A. Parlett, Olga Bortolini, Mark A. Isaacs, Alessandro Massi, Carmine D’Agostino

**Affiliations:** † Department of Chemical, Pharmaceutical and Agricultural Sciences, University of Ferrara, Via L. Borsari 46, 44121 Ferrara, Italy; ‡ Department of Chemical Engineering, University of Manchester, Oxford Road, Manchester M13 9PL, U.K.; § Diamond Light Source, Harwell Science and Innovation Campus, Didcot OX11 0DE, Oxfordshire, U.K.; ∥ Catalysis Hub, Research Complex at Harwell Rutherford Appleton Laboratory, Harwell OX11 0FA, Oxfordshire, U.K.; ⊥ Department of Environmental and Prevention Sciences, University of Ferrara, Via L. Borsari 46, 44121 Ferrara, Italy; # Department of Chemistry, University College London, London WC1H 0AJ, U.K.; ¶ HarwellXPS, Research Complex at Harwell, RAL, Didcot OX11 0FA, U.K.; ∇ Dipartimento di Ingegneria Civile, Chimica, Ambientale e dei Materiali (DICAM), Università di Bologna (UNIBO), via Terracini n. 28, 40131 Bologna, Italy

**Keywords:** humins, furfuryl alcohol, NMR relaxation, diffusion, SBA-15-based catalysts, heterogeneous catalysis, biobased molecules

## Abstract

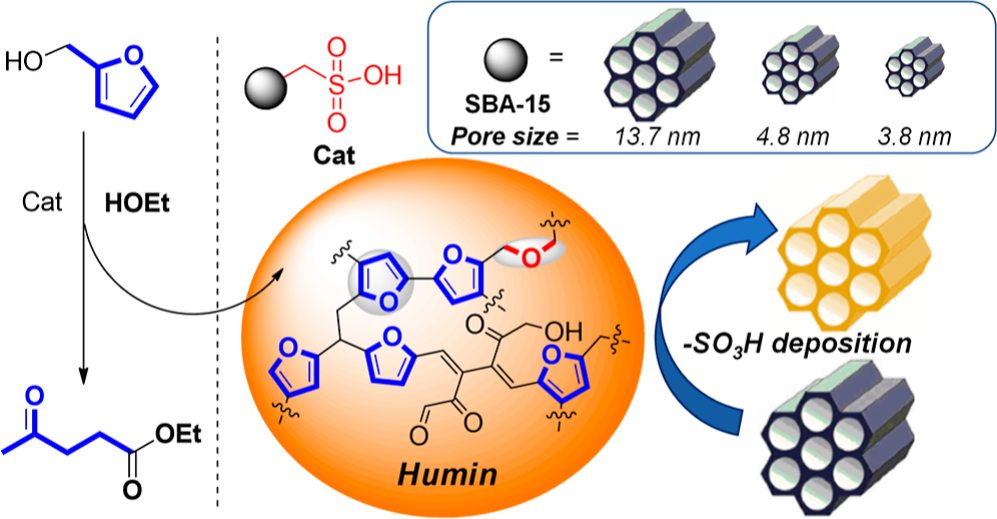

Herein, the alcoholysis of furfuryl alcohol in a series of SBA-15-pr-SO_3_H catalysts with different pore sizes is reported. Elemental analysis and NMR relaxation/diffusion methods show that changes in pore size have a significant effect on catalyst activity and durability. In particular, the decrease in catalyst activity after catalyst reuse is mainly due to carbonaceous deposition, whereas leaching of sulfonic acid groups is not significant. This effect is more pronounced in the largest-pore-size catalyst **C3**, which rapidly deactivates after one reaction cycle, whereas catalysts with a relatively medium and small average pore size (named, respectively, **C2** and **C1**) deactivate after two reaction cycles and to a lesser extent. CHNS elemental analysis showed that **C1** and **C3** experience a similar amount of carbonaceous deposition, suggesting that the increased reusability of the small-pore-size catalyst can be attributed to the presence of SO_3_H groups mostly present on the external surface, as corroborated by results on pore clogging obtained by NMR relaxation measurements. The increased reusability of the **C2** catalyst is attributed to a lower amount of humin being formed and, at the same time, reduced pore clogging, which helps to maintain accessible the internal pore space.

## Introduction

Since the industrial revolution, fossil fuels (oil, natural gas, and coal) have been the predominant feedstock for the production of chemicals and energy.^1^ However, their inherent nonrenewability and impact on climate change due to the release of greenhouse gases have become a global concern.^2^ As a result, a shift to more sustainable, renewable resources and processes is paramount. Due to the Renewable Energy Policy Network for the 21st century (REN21), the energy supply from renewable resources has increased rapidly in recent years,^3^ primarily from hydrothermal, wind, and solar power. However, the renewable and sustainable manufacture of chemical commodities remains challenging, with the vast majority of the chemical industry utilizing raw materials derived from fossil fuel-based materials.^2,4^ Therefore, it is increasingly important to develop alternative synthetic routes to existing key chemicals or develop sustainable substitutes.^5,6^

Lignocellulosic biomass is one of the most prominent and promising renewable feedstocks for the production of fuels and platform chemicals, consisting predominately of cellulose, hemicelluloses, and lignin. One key advantage of lignocellulosic biomass is that it is produced from nonedible parts of food crops, so it does not compete with food production. Valorization of lignocellulosic biomass begins with biorefinery processing of lignocellulose, which consists of several physical and chemical pretreatments to deconstruct the feedstock into cellulose, hemicellulose, and lignin.^7^ Cellulose and hemicellulose can undergo further processing, *via* saccharification, to yield C6 and mixtures of C6 and C5 sugars, respectively. In turn, C6 and C5 sugars can be used to produce a vast array of important platform chemicals used in the production of a wide variety of high-value chemicals, fuels, solvents, bioplastics, and resin monomers.^8−10^

**Scheme 1:**
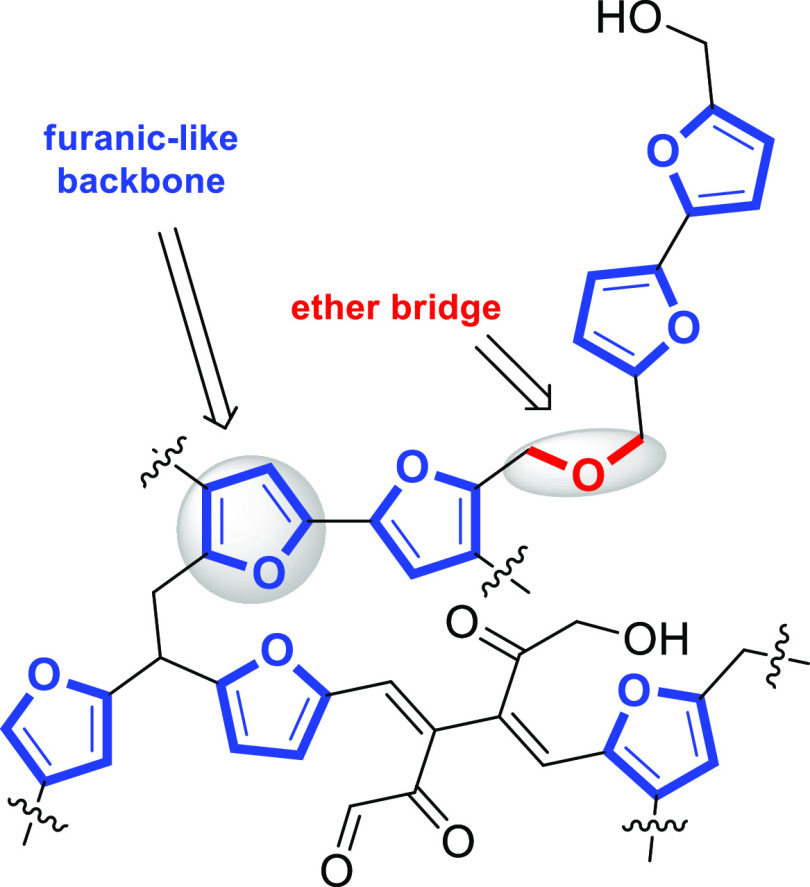
Generic Structure of Humins Formed during Alcoholysis/Hydrolysis of FOL and 5-HMF

Pentose-derived furfural (FUR) and hexose-derived 5-hydroxymethylfurfural (5-HMF) are highly desirable platform chemicals owing to the inherent reactivity of their functional groups (hydroxy, double bond, and carboxyl groups) that can be exploited to obtain molecules with different properties *via* well-known chemical transformations.^11,12^ FUR, derived from pentose saccharides *via* dehydration, has a projected market value of €500 million by 2024.^13−16^ Furfuryl alcohol (FOL), tetrahydrofuran, 2-methyltetrahydrofuran and maleic anhydride, all obtainable from FUR, are among those molecules of great academic and industrial interest.^17^ FOL is of particular interest due to its applications as a fuel component and polymer precursor.^18^ A wide range of methods for the reduction of FUR to FOL have been reported, with a significant number employing catalytic carbonyl hydrogenation over palladium,^19^ copper,^20^ and zirconium, the latter *via* the Meerwein–Ponndorf–Verley reduction.^21^ FOL can be further transformed into levulinic acid (LA) and its ester analogues, such as ethyl levulinate (EL), which are fundamental to producing a range of biobased fuels, solvents, and polymers. Both are easily obtained by either homogeneous or heterogeneous FOL hydrolysis/alcoholysis catalyzed by Brønsted acids.^22−25^ However, such processes tend to result in unwanted side reactions, including the acid-catalyzed polymerization of reactive species to unwanted oligomers, known as humins, representing a major drawback in these reactions.^26^ Humins are composed of a mixture of highly polydispersed oligomeric/macromolecular byproducts, comprising a furanic-like backbone held together by short aliphatic chains and ether bridges. Higher-molecular-weight fractions are generally insoluble in both organic and aqueous solvents (Scheme 1).

Thus, while the formation of humins negatively impacts the overall product yield, it simultaneously further complicates product isolation. Typically, this is very energetically or chemically demanding for homogeneous systems, leading to considerable waste generation from acid quenching and washing. Minimizing humin formation has been achieved to a degree *via* fine control over process conditions, *i.e.*, temperature, concentration, cosolvent ratio, and pH.^27−30^ Nonetheless, their formation remains a major limitation for scale-up and industrial production of valuable platform chemicals.

As such, the development of heterogeneous catalytic systems is greatly desirable in the industrial sector owing to the possibility of recycling the catalyst and simplifying purification/separation steps by simple filtration of the catalyst from the reaction mixture.^31−33^ Unfortunately, humin formation is a great concern for heterogeneous catalytic systems when compared to homogeneous systems due to the deposition of the insoluble, carbonaceous, macromolecular material over the catalyst surface and within the catalyst pores, resulting in deactivation of the catalyst active sites.^34^ Therefore, it is clear that unraveling the effect of humin formation and its role in heterogeneous catalyst deactivation is important in order to aid a more efficient design of such systems. However, this aspect of such critical chemical processes has remained relatively unexplored.^35^

Herein, a study combining various analytical techniques, including elemental analysis (EA), IR, solid-state NMR, and N_2_ adsorption–desorption analysis, with NMR relaxation and diffusion techniques is reported. The impact of humin formation and deposition upon heterogeneous catalyst deactivation during the Brønsted-acid-catalyzed alcoholysis of FOL to EL, mediated by a series of SBA-15-pr-SO_3_H catalysts with tuned mesopore size, is thereby thoroughly investigated and described.

## Results and Discussion

### Catalyst Activity and Stability Tests

To evaluate the role of the catalyst pore diameter on catalyst deactivation *via* humin accumulation during the FOL esterification over silica-supported sulfonic acid catalysts, three SBA-15 supports with tuned mesopore diameter were synthesized.^36,37^ By controlling the hydrothermal treatment temperature, catalysts with average pore sizes ranging from 3.8 to 13.7 nm were prepared. The main textural properties (surface area, pore volume, and average pore diameter) obtained from N_2_ adsorption–desorption analysis of the three SBA-15 architectures are summarized in Table 1 (denoted **S1** for small, **S2** for medium, and **S3** for large SBA-15 materials). Both the average pore diameter and pore volume correlate positively with the hydrothermal treatment temperature, while the Brunauer–Emmett–Teller (BET) surface area effectively remains constant.

**Table 1:** Structural Properties of Synthesized SBA-15 Silicas Produced by Varying the Hydrothermal Temperature^a^ Used during Catalyst Preparation

SBA-15	preparation temperature (°C)^b^	BET surface area (m^2^/g)^c^	pore volume (mL/g)^d^	average pore diameter (nm)^d^
**S1**	50	752 ± 75	0.70 ± 0.07	3.8 ± 0.4
**S2**	80	761 ± 76	0.81 ± 0.08	4.8 ± 0.5
**S3**	120	686 ± 69	2.89 ± 0.29	13.7 ± 1.4

a Reaction conditions reported in the Experimental Section.

b Hydrothermal treatment temperature.

c Calculated by BET analysis.

d Calculated by BJH anaysis using the desorption branch.

**Figure 1:**
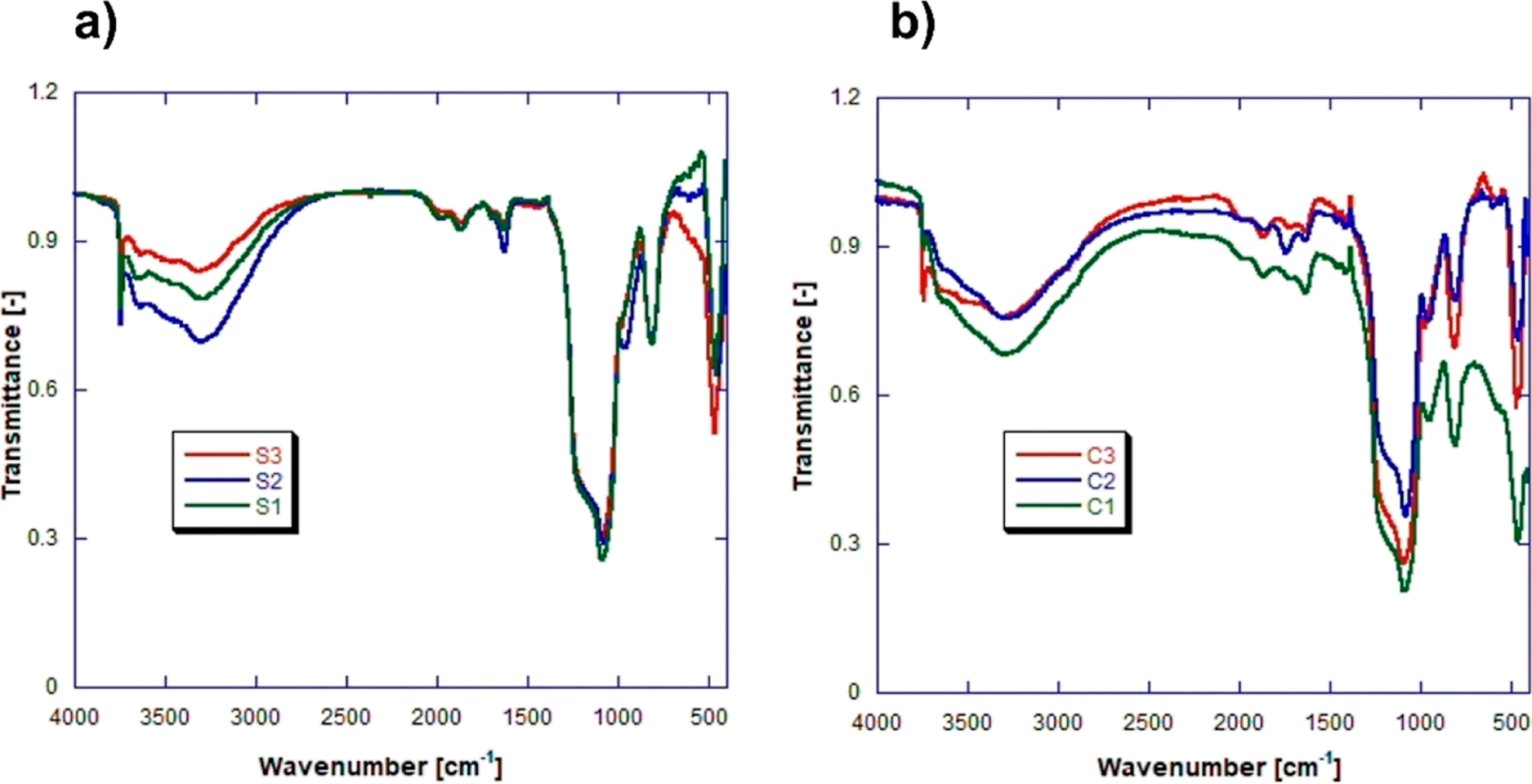
FT-IR spectra of the (a) SBA-15 silica supports and (b) functionalized SBA-15-pr-SO_3_H catalysts.

The sulfonic acid functionality was introduced onto the SBA-15 support *via* grafting of (3-mercaptopropyl)trimethoxysilane (3-MPTMS) and subsequent thiol oxidation using H_2_O_2_ and sulfuric acid. The complete conversion of the thiol group into −SO_3_H was confirmed by the absence of the thiol stretching frequency (2550–2600 cm^–1^) in the Fourier transform infrared (FT-IR) spectra as reported in Figure 1b.

**Figure 2:**
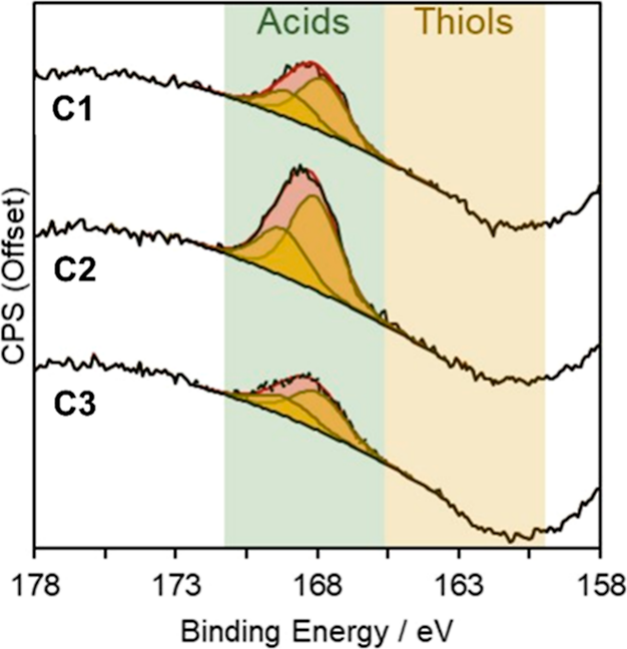
S 2p XPS spectra for the fresh catalysts deconvoluted to the 3/2 and 1/2 doublet expected.

To further confirm the nature of sulfur speciation, specifically, the complete oxidation of the thiol (SH) functionality to sulfonic acid (−SO_3_H) groups by H_2_O_2_ treatment, S 2p X-ray photoelectron spectroscopy (XPS) was conducted, as shown in Figure 2. We can rule out any residual thiol from the absence of a peak centered at ∼164 eV, while the peak with a binding energy of ∼169 eV is consistent with that for the sulfonic acid functionality, with all three catalysts displaying only the single doublet of sulfonic acid.

The resulting loadings, determined *via* EA (see the Supporting Information for details) and titration, are comparable across the three supports with equal surface acid site density, as shown in Table 2. This suggests a similar surface −OH density across the different silica supports and therefore an equal number of grafted sites.

**Table 2:** Loading of SBA-15-pr-SO_3_H-Functionalized Silicas

SBA-15-pr-SO_3_H	catalyst loading (mmol/g)^a^	acid capacity (mmol [SO_3_H]/g [catalyst])^b^
**C1**	0.38 ± 0.01	0.37 ± 0.02
**C2**	0.43 ± 0.01	0.46 ± 0.02
**C3**	0.36 ± 0.01	0.36 ± 0.02

a Catalyst loading calculated by EA through sulfur percentage measurements in the sample.

b Acid capacity calculated by titration.

**Figure 3:**
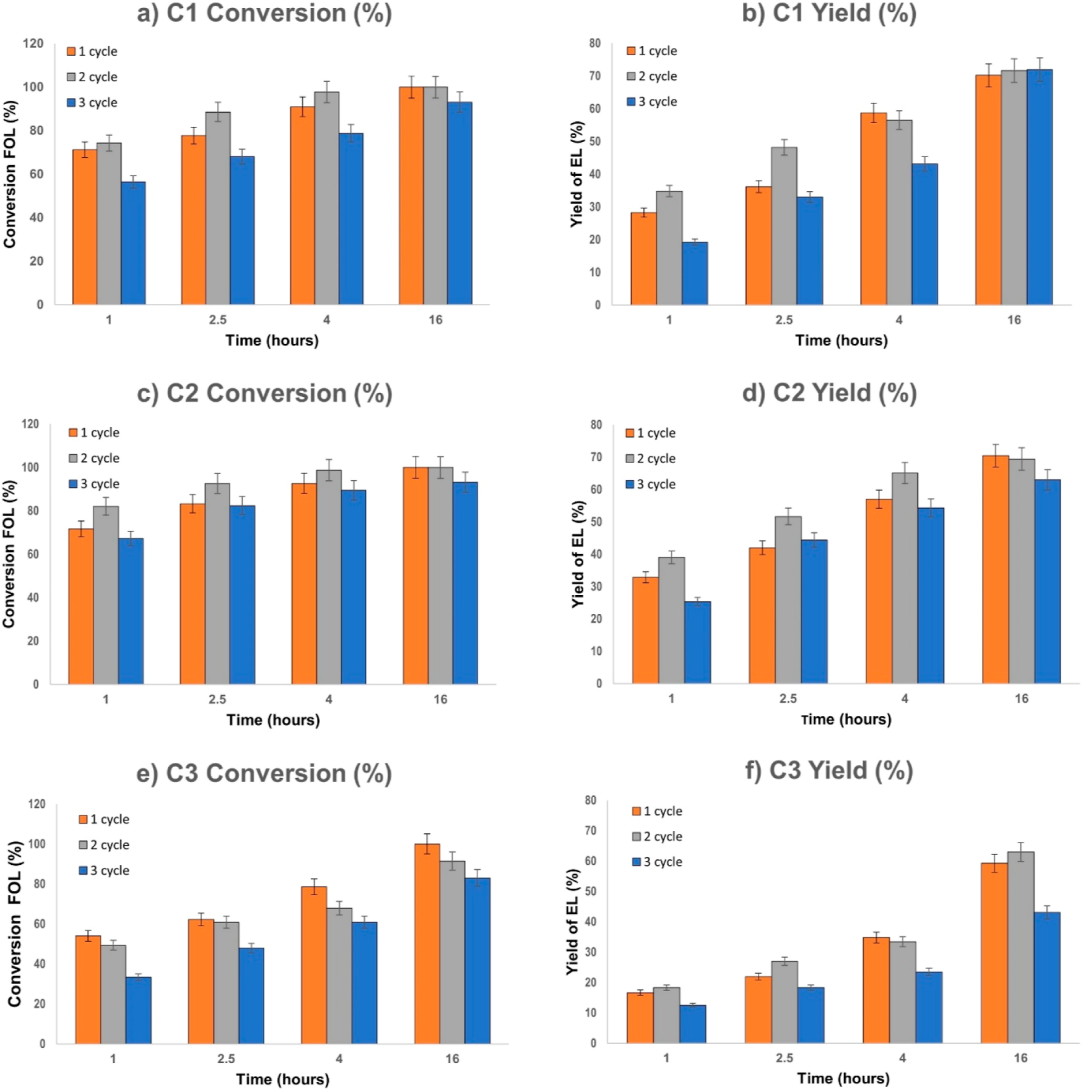
Conversion of FOL and yield of EL for **C1** small, **C2** medium, and **C3** large SBA-15-pr-SO_3_H catalysts. **C1**, **C2**, or **C3** (100% w/w) was suspended in a solution of FOL (0.3 M) in EtOH and stirred at 120 °C. Conversion *vs* time (a,c,e) and yield *vs* time (b,d,f) bar charts were obtained through GC analysis.

**Scheme 2:**
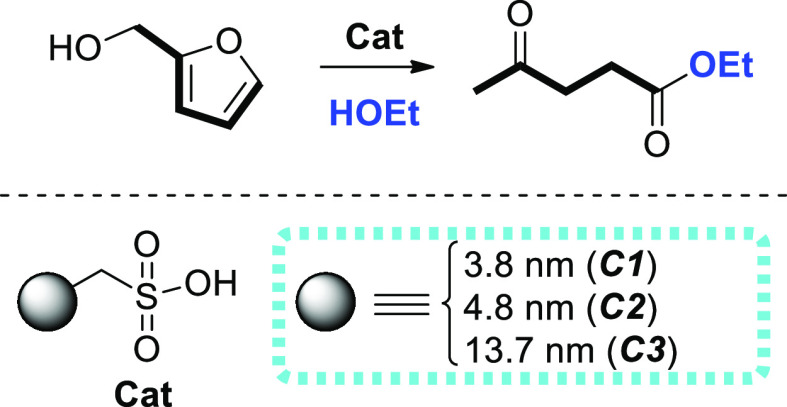
Alcoholysis of FOL to EL in EtOH Mediated by SBA-15-pr-SO_3_H (**C1**, **C2**, or **C3**)

**Scheme 3:**
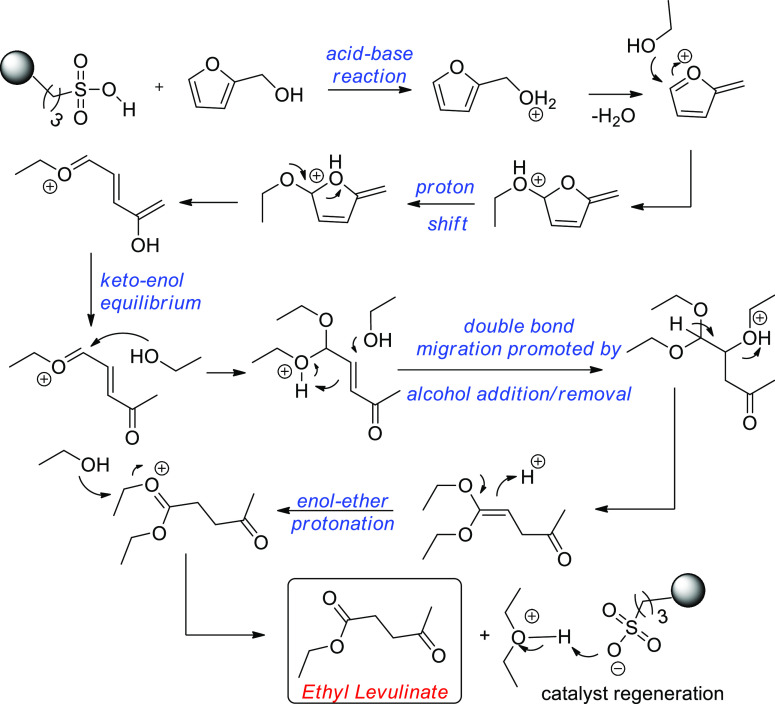
Proposed Mechanism for Acid-Catalyzed Alcoholysis of FOL to EL in EtOH

The performance of the sulfonic acid catalysts was evaluated for the alcoholysis of FOL to EL (Scheme 2). The catalyst acts as a proton source by dissociation of sulfonic acid. Alcoholysis of FOL occurs in several steps under these conditions. A proposed mechanism is depicted in Scheme 3 according to the studies of Dumesic *et al.*(38) Reaction profiles illustrating conversion of FOL and yield of EL as a function of time for the three catalysts, along with data from two recycle runs to evaluate potential losses in activity, are reported in Figure 3. Between reaction cycles, spent catalysts were washed several times with ethanol as described in the Experimental Section to remove the soluble, low-molecular-weight fraction of humins.

For the first cycle of experiments, the catalyst performance decreases slightly with increasing pore size of the SBA-15 support. In fact, **C1** and **C2** mesopores lead to an average reaction rate *R* of 5.8 × 10^–3^ s^–1^ (normalized to −SO_3_H concentration) and 5.4 × 10^–3^ s^–1^, respectively, calculated at 1 h. Conversely, FOL conversion over the large mesopore catalyst is lower at the same point (*R* of 4.8 × 10^–3^ s^–1^). *R* values observed in our experiments are in line with those reported in the literature (*i.e.*, the alcoholysis reaction of FOL in *n*-butanol at 110 °C with SBA-15-pr-SO_3_H, reported by Pagliaro *et al.*, has an *R* of 3.0 × 10^–3^ s^–1^).^39^ Across the three systems, the conversion (consumption of FOL) and product yield (formation of EL) differ, mainly due to the formation of humin.^40^ For the first reuse of the catalysts (cycle 2), an appreciable drop in reactivity is observed only for large-pore-size SBA-15-pr-SO_3_H. In contrast, a decrease in performance (both conversion and yield) is seen across all catalysts for the second reuse (cycle 3), even though **C1** and **C2** SBA-15-pr-SO_3_H catalysts exhibited higher performance in terms of conversion and yield compared to the larger-pore SBA-15-pr-SO_3_H catalyst **C3**. This significant decrease in catalyst performance after each reuse for the larger-pore-size catalyst suggests a more rapid accumulation of humins within the porous framework. EL yields at 100% FOL conversion further support this conclusion, with lower yields for **C3**.

To understand the reasons for catalyst deactivation with reuse, the possibility of leaching of the organic moiety [Si(OMe)-pr-SO_3_H], as well as clogging due to humin deposition were assessed by CHNS EA. Leaching of the active site has been calculated by measuring the active site loading after each cycle, as shown in the Supporting Information (see eqs S24 and S25) and the results are summarized in Table 3. Surface EA, from XPS, is reported in Tables S1 and S2, and shows equivalent, albeit slightly higher (due to the surface sensitivity of the technique) increases in C content with each recycle for the three pore-size catalysts.

**Table 3:**
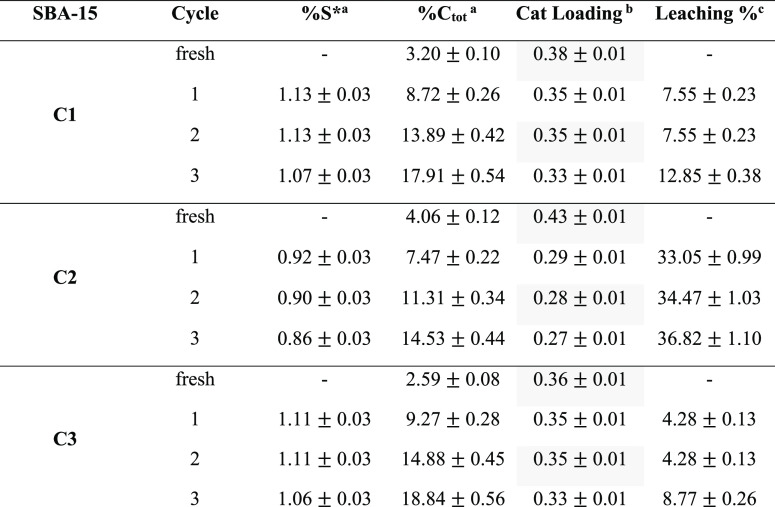
Leaching Data for the Spent Catalysts

a Genuine percentage of sulfur (%S^*^) calculated using eq S24 reported in the Supporting Information.

b Catalyst loading as mmol of Si(OMe)-pr-SO_3_H *per* gram (mmol g^–1^), see eq S25 in the Supporting Information.

c Percentage of active sites [Si(OMe)-pr-SO_3_H] leached with respect to the fresh catalyst. Percentages were measured by CHNS EA.

**Figure 4:**
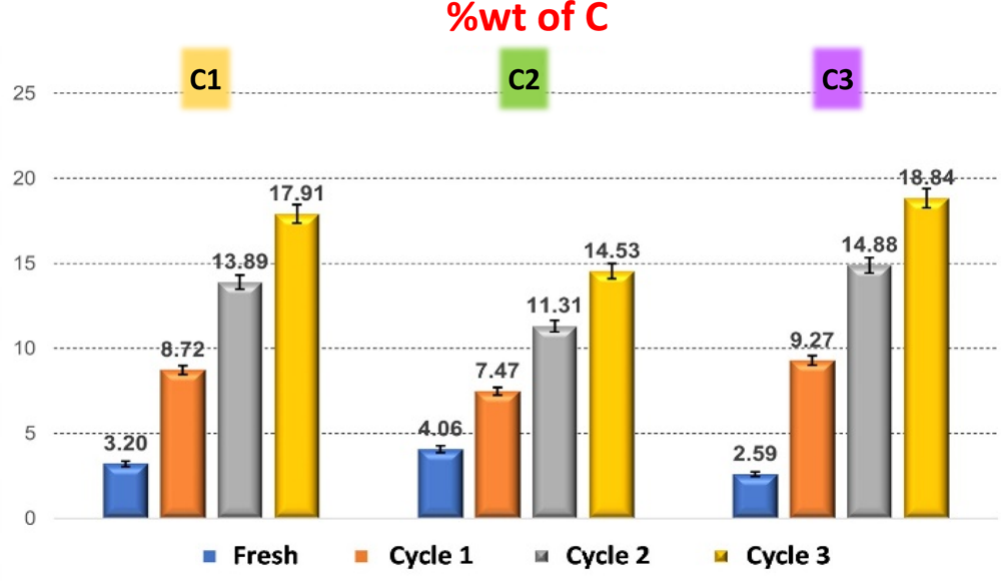
Weight percentage of carbon detected by CHNS EA during subsequent experiment cycles of **C1**, **C2**, and **C3** pore catalysts.

Interestingly, the medium-pore-size SBA-15-pr-SO_3_H catalyst shows greater leaching of sulfur after the first use than both the small- and large-pore-size catalysts, while the large-pore-size catalyst shows less leaching of sulfur than the small-pore-size catalyst. Furthermore, according to the kinetic experiments reported in Figure 3, the medium-pore-size catalyst shows no drop in activity after one cycle, whereas the large-pore-size catalyst shows a drastic decrease, despite negligible leaching. The results reported in Table 3 suggest that leaching is not the main cause of deactivation; therefore, it is reasonable to assume that the deposition of humins during the reaction, as observed by CHNS EA (Figure 4) and XPS (Tables S1 and S2), is the main cause of catalyst deactivation.^41^

Carbon content from EA of the fresh and spent catalyst reveals significant amounts of carbon (C) deposition, which is attributed to humin formation. The initial increase is lower in both **C1** and **C2** catalysts (5.5 and 3.3% increase in % C from fresh to cycle 1, respectively) and larger for **C3** (6.7% increase in % C from fresh to cycle 1). Further increases in humin deposition are relatively consistent for **C2** (3.2–3.8% increase in % C per cycle), while **C1** and **C3** experience a larger increase in carbonaceous deposition after cycle 2 (5.2 and 5.6% increase in % C for cycle 2, respectively) than after cycle 3 (both *ca.* 4% increase in % C for cycle 3). The increase in carbon accumulation and decreasing catalytic performance with an increasing number of cycles suggest a significant impact of humin deposition upon the accessibility to the catalytic site, which inhibits the conversion to both desirable EL and unwanted humins. The amount of carbon deposited is greater in **C1** and particularly in **C3**, which agrees well with the catalyst stability tests reported in Figure 3, showing a more significant deactivation for **C1** and particularly **C3**.

**Figure 5:**
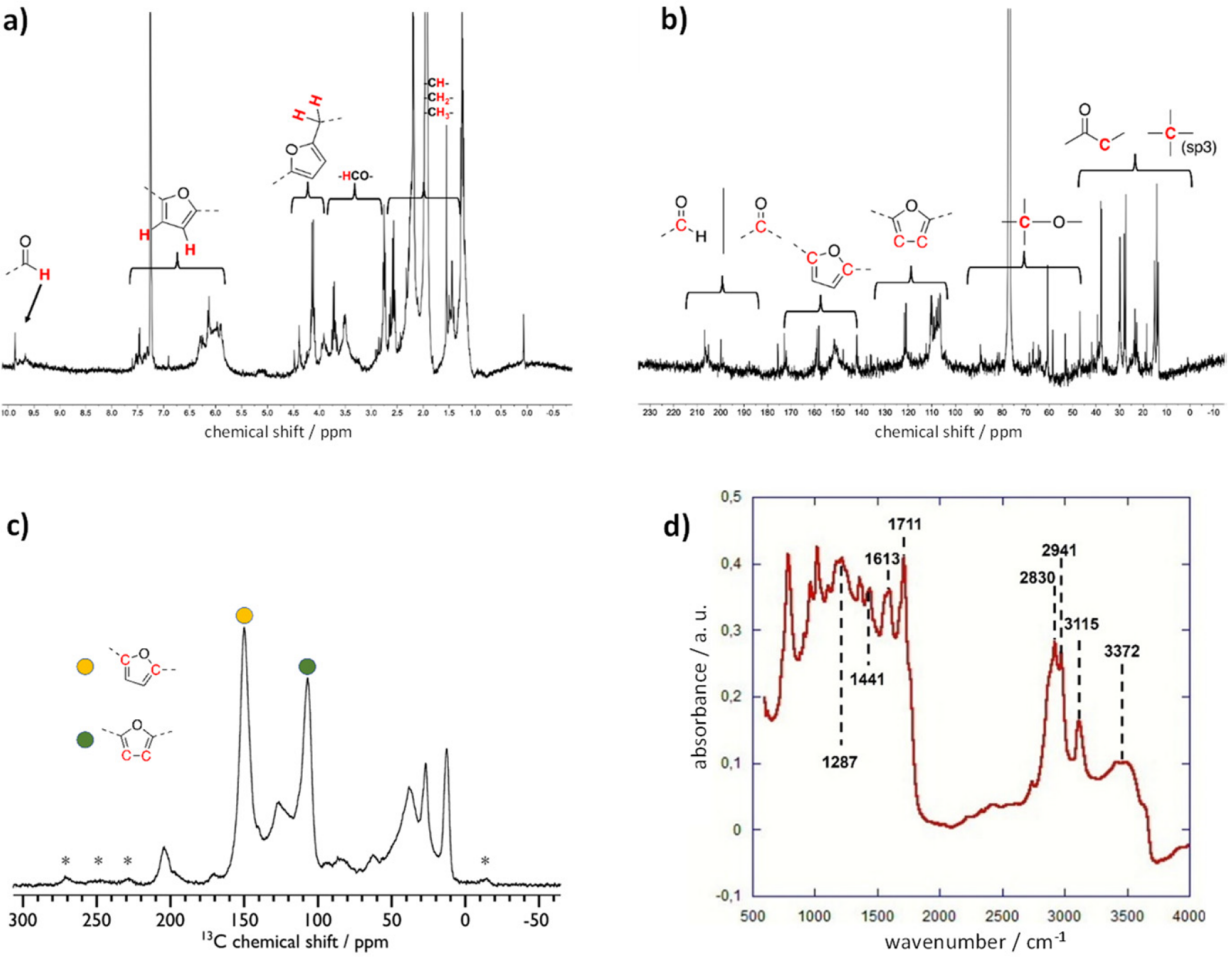
(a) ^1^H NMR spectrum of soluble humin fractions in CDCl_3_ extracted with 5 mL of acetone. (b) ^13^C NMR spectrum of soluble humin fractions in CDCl_3_ extracted with 5 mL of acetone. (c) {^1^H-}^13^C CPMAS NMR spectrum of solid humin, isolated from the catalyst *via* NH_4_HF_2_ treatment. Spinning side bands are denoted with asterisks. (d) DRIFTS spectrum of solid humin, isolated from the catalyst *via* NH_4_HF_2_ treatment.

Characterization of humin has been carried out by NMR analysis and DRIFTS. Humins are formed as highly polydisperse polymers in which lower-molecular-weight fractions are commonly soluble in organic solvents. ^1^H and ^13^C NMR spectra of the fractions soluble in acetone are reported in Figure 5a,b.

The main functional groups of humins are clearly present on both spectra: the aldehyde singlets (between 9.72 and 9.54 ppm in the ^1^H spectrum and between 210 and 196 ppm in the ^13^C spectrum), the aromatic protons of furan rings (between 7.5 and 5.9 ppm), and the protons (and carbon) attached to carbons directly bound to furans and oxygen (the region of singlets from 4.5 to 3.0 ppm in ^1^H and the region from 91 to 50 ppm in ^13^C). These results are in line with the literature.^42^

Furthermore, the nonsoluble fractions of humin were isolated by treating the spent catalyst **C2** with NH_4_HF_2_ and the sample was analyzed by solid-state NMR spectroscopy and DRIFTS. The ^13^C NMR spectrum of the solid, shown in Figure 5c, demonstrates the formation of rigid humins. Several functional groups can be recognized, in particular, furanic carbon at the α position (150 ppm) and furanic carbon at the β position (110 ppm).^43,44^ Finally, the DRIFTS spectrum (Figure 5d) clearly shows the presence of the main functional groups (Table 4) belonging to the generic humin structure.^45^

**Table 4:** Characteristic Wavenumbers of the DRIFTS Spectrum of the Insoluble Fraction of Humin

wavenumber (cm^–1^)	assignment
3372	ν(OH)
3115	ν(CH sp^2^)
2941	ν(CH sp^3^)
2830	
1711	ν(C=O)
1613	
1441	ν(C=O) furan ring
1287	ν(CO)

**Scheme 4:**
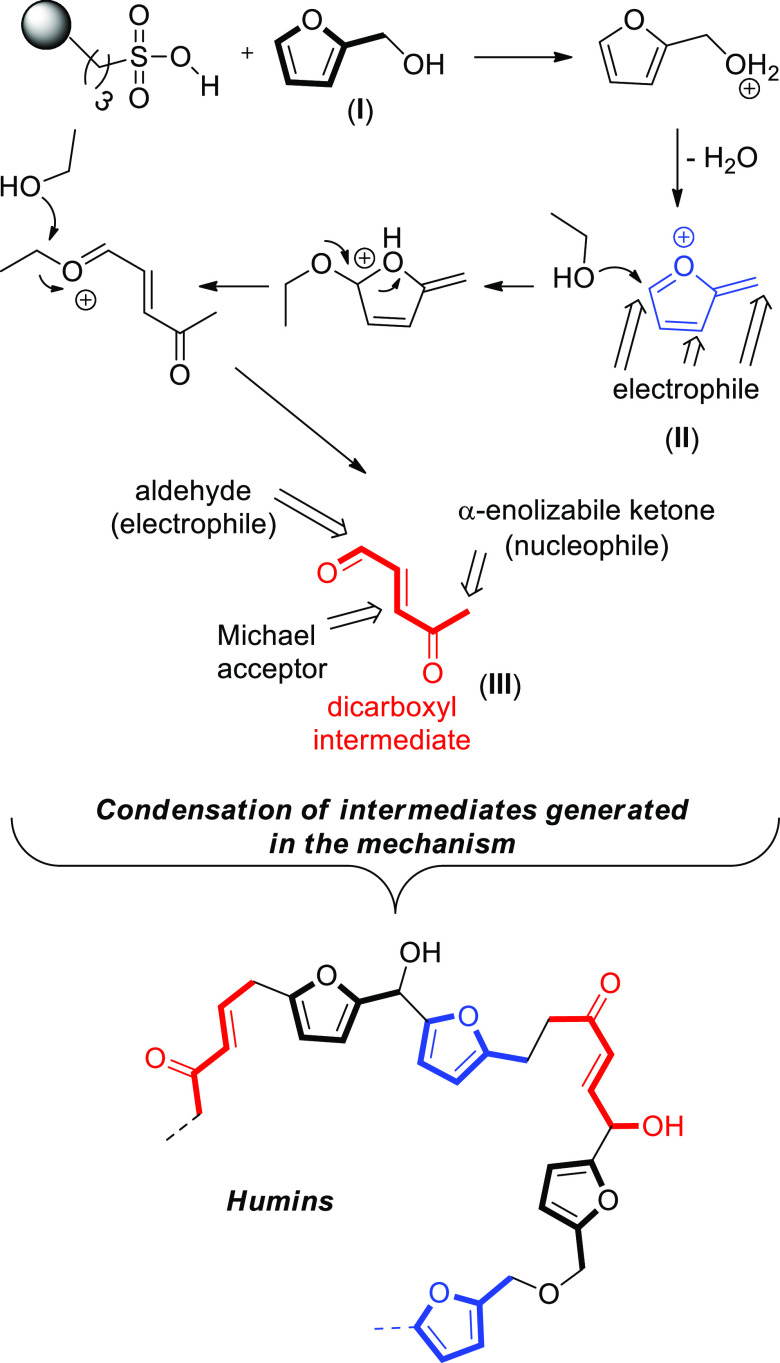
Proposed Mechanism for the Uncontrolled Polymerization of the Intermediates of Acid-Catalyzed Alcoholysis of FOL to EL in EtOH

A proposed mechanism for the uncontrolled formation of humins by reactions among the intermediates formed in the acid-catalyzed alcoholysis of FOL is depicted in Scheme 4.

### Diffusion and Adsorption Studies

To further understand the effect of humin deposition on diffusion and adsorption properties within catalysts with different pore sizes, NMR diffusion and relaxation studies were performed. NMR relaxation measurements have previously been demonstrated to be a powerful, nondestructive method to probe the interactions of molecules over surfaces of inorganic/organic heterogeneous catalysts.^46−51^ In addition, pulsed-field gradient (PFG) NMR experiments can be used to measure diffusion coefficients in a variety of situations, including liquid mixtures,^52,53^ ionic solvents,^54^ liquids imbibed within porous materials,^55−57^ and to probe accessibility of molecules in pore structures.^58,59^

**Figure 6:**
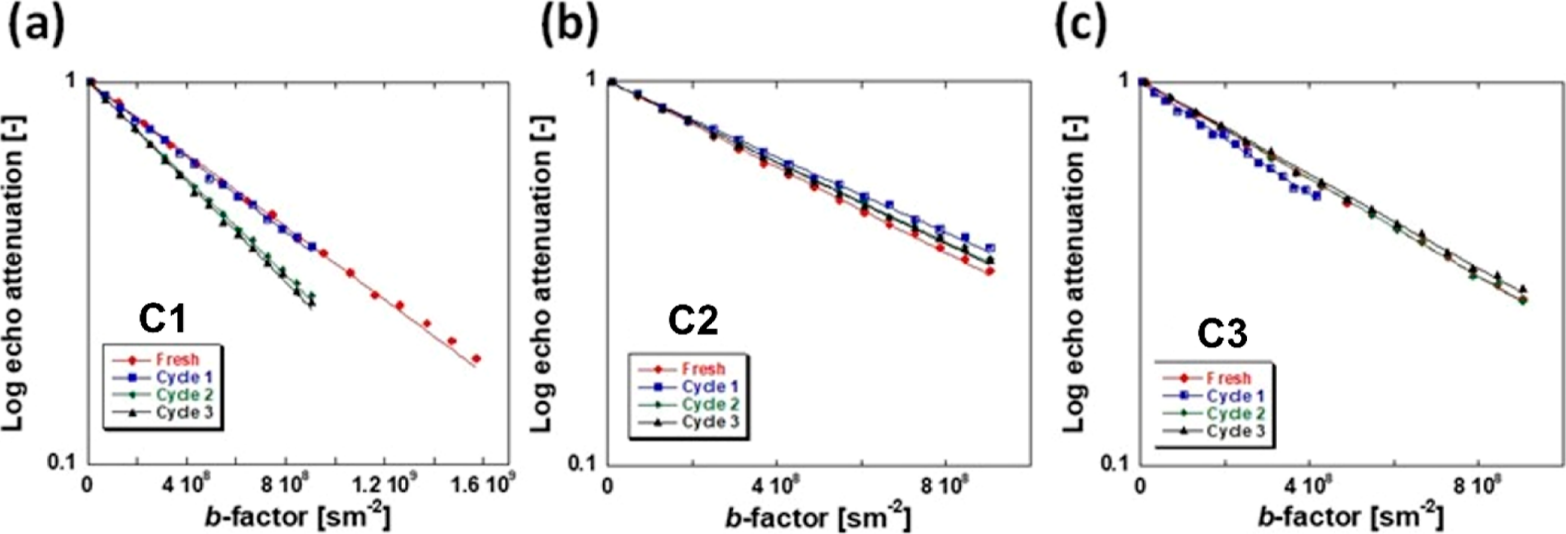
PFG NMR attenuation plots for *n*-octane imbibed within (a) **C1**, (b) **C2**, and (c) **C3** catalysts after various cycles of reuse.

Figure 6 shows the PFG NMR log attenuation plots of the *n*-octane probe molecule imbibed within the pores of the different catalysts. Specific details of the PFG NMR method and experiments performed are given in the Supporting Information.

**Figure 7:**
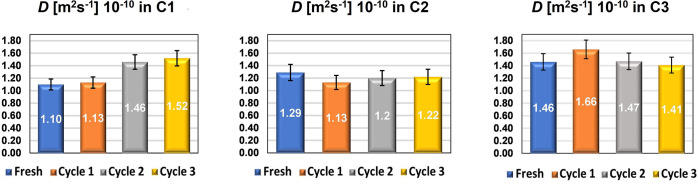
Diffusion coefficients of *n*-octane in fresh and recycled **C1** (left), **C2** (middle), and **C3** (right) catalysts. Error bars represent a relative error in the range of 5–6%.

The self-diffusion coefficients *D* obtained from the plots in Figure 6 are reported in Figure 7.

The self-diffusion coefficient within the pores of the fresh catalyst increases moving from smaller- (**C1**) to large-pore-size (**C3**) SBA-15 catalysts. A similar behavior has previously been observed in porous oxides when introducing larger pores into the framework.^58^ The mobility inside the pores as a function of cycles of reuse remains almost the same for **C2** and **C3**, whereas **C1** shows an increase in diffusivity after each reuse. While the latter result may seem counterintuitive, it is reasonable to assume that because of the relatively small pore size, the pathways with higher tortuosity, that is, lower average molecular displacement, are being made less accessible to molecules due to pore restrictions caused by humin deposits. This will occur more easily in such small pores, thereby facilitating diffusion through alternative pathways of lower tortuosity (*i.e.*, larger pores with better connectivity), similar to what has been previously observed inside hierarchical porous materials.^59^ We note here that the tortuosity of a porous medium is inversely proportional to the self-diffusion coefficient measured by PFG NMR using a weakly interacting probe molecule such as *n*-octane; that is, a lower self-diffusivity indicates a higher degree of tortuosity. Therefore, it can be deduced that, for the small-pore catalysts, the porous network becomes less tortuous with increasing reuses likely due to pore blockage by humin deposition of the smallest pores interconnecting the porous network. This is also supported by porosimetry data on spent catalysts (see Table S3 of the Supporting Information), which shows that, for **C1**, there is a significant reduction of surface area and pore size after each reuse, which is relatively large when compared to the behavior of **C2** and **C3**. This suggests that the deactivation mode of the small-pore catalyst is different from that occurring in catalysts with larger pores, whereby in the latter, changes in textural properties with catalyst reuse are also observed but to a smaller extent compared to the small-pore catalyst.

For **C2** and **C3**, humin deposition does not have a significant effect on the catalyst self-diffusivity/tortuosity; hence, the rate of bulk to pore diffusion is not significantly affected after each cycle of reuse. That is, humin deposition in **C2** and **C3** does not significantly affect the tortuosity of the pore structure. This suggests that humin deposition over the catalyst surface does not lead to significant pore blockage; rather, it covers the surface of the internal pore space but without blocking the pores. The results also suggest that for larger pores, catalyst deactivation caused by humin deposition does not occur *via* diffusion limitation.

**Figure 8:**
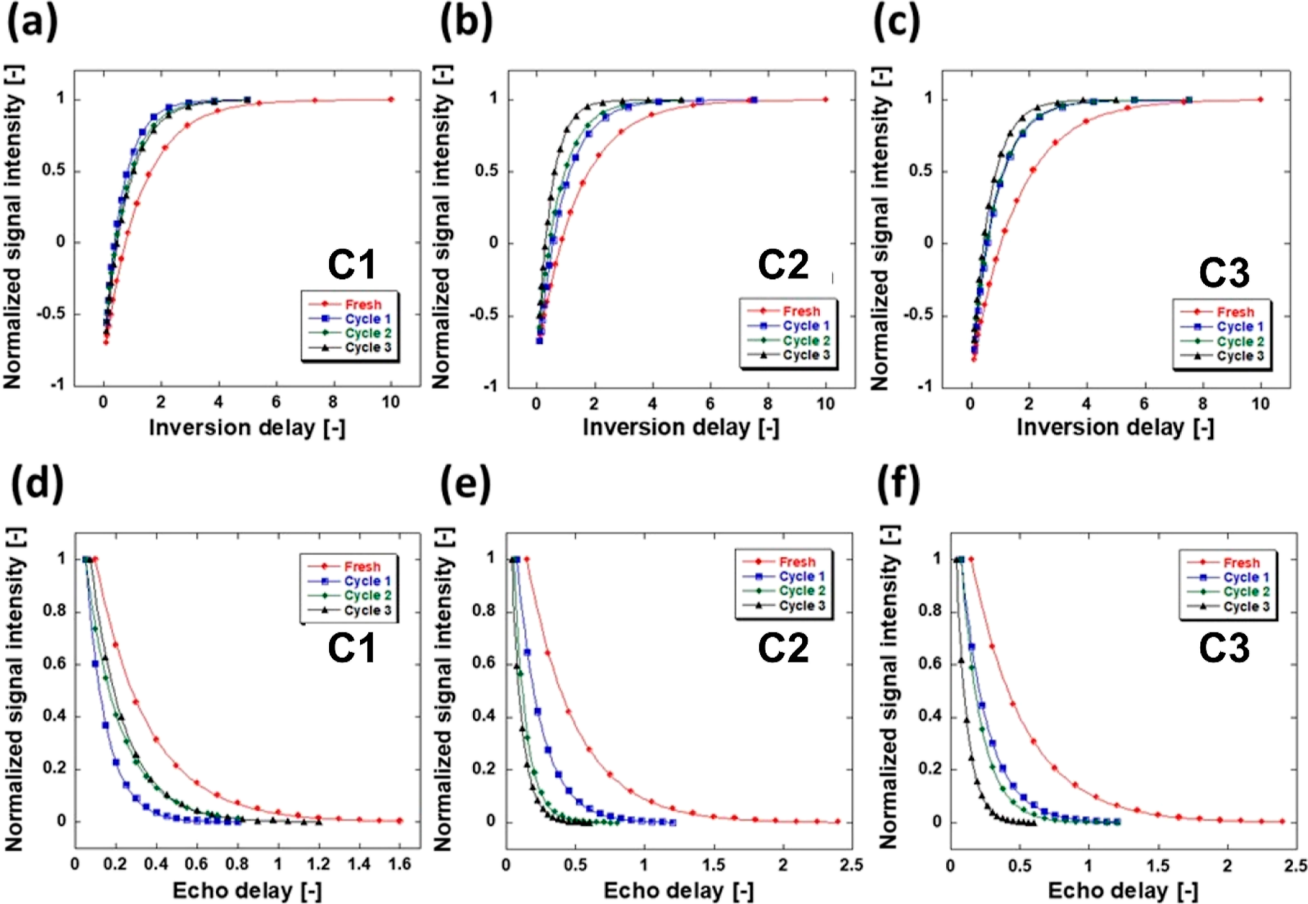
(a–c) *T*_1_ inversion recovery and (d–f) *T*_2_ CPMG plots obtained using *n*-octane imbibed within the pores of the fresh and recycled **C1**, **C2**, and **C3** catalysts.

NMR relaxation experiments were performed to probe the surface of the spent catalyst. Deposition of organic carbonaceous deposits should result in a more lipophilic surface with an increased affinity toward apolar molecules. The ratio of the spin–lattice relaxation time (*T*_1_) and the spin–spin relaxation time (*T*_2_) measured by NMR relaxation techniques can be correlated with the strength of adsorbate/adsorbent surface interactions where an increase of *T*_1_/*T*_2_ generally indicates a stronger interaction with the surface.^60−62^*T*_1_ values of *n*-octane in the fresh and spent catalysts are measured using the inversion recovery sequence and *T*_2_ values by using the Carr–Purcell–Meiboom–Gill (CPMG) pulse sequence (see the Supporting Information for details); the resultant plots are reported in Figure 8.

**Figure 9:**
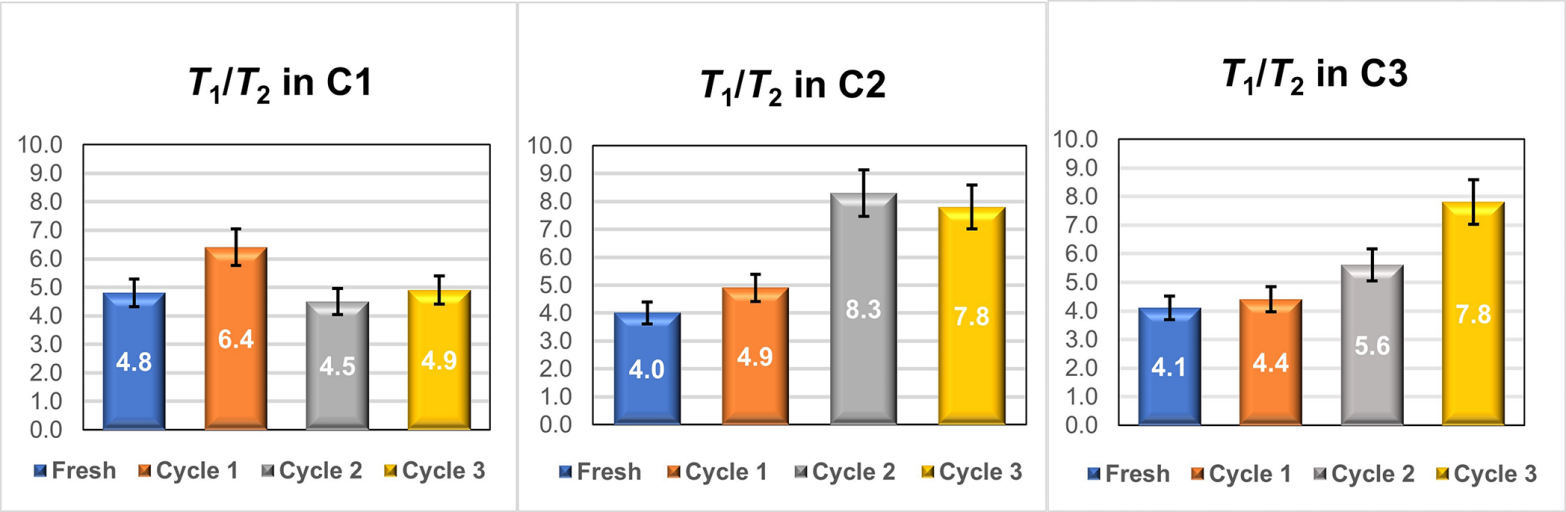
*T*_1_/*T*_2_ ratio of *n*-octane imbibed in fresh and recycled **C1** (left), **C2** (middle), and **C3** (right) catalysts. Error bars represent a relative error in the range of 5–6%.

*T*_1_/*T*_2_ values obtained are reported in Figure 9. For the small-pore catalyst, *T*_1_/*T*_2_ of *n*-octane increases after the first reuse of the catalyst (cycle 1), which can be explained by the interactions with lipophilic humin deposits formed inside the catalyst pores, which makes the surface more hydrophobic compared to the fresh catalyst. *T*_1_/*T*_2_ then decreases after reusing the catalyst a second (cycle 2) and third (cycle 3) time. This can be attributed to blockage of the internal pores after the first reuse, as suggested by the diffusion data, which prevents *n*-octane from diffusing within the innermost part of the internal pore network. Hence, *n*-octane mostly probes the hydrophilic external surface, which is less affected by humin deposits. Indeed, the behavior of sulfonic acid groups could be affected by their location, for example, by differences in geometric confinement. A similar behavior has previously been reported by silanol groups in zeolites.^63^ One possible reason for this observation is that confinement effects play a significant role in determining humin formation; that is, in more confined spaces the rate of humin formation can be enhanced.

For **C2** and **C3**, there is instead a general increase of *T*_1_/*T*_2_ of *n*-octane with subsequent reuses. This can be explained by the increasing amount of humin deposits on the catalyst surface with increasing reuses. However, unlike **C1**, the larger pore size of the medium and large-pore catalysts does not lead to a complete blockage of the internal pore network. Rather, there is a buildup of carbonaceous deposits on the pore walls, which remains accessible albeit with reduced pore dimensions and shielding of the active sites. Hence, *n*-octane molecules are still able to diffuse inside the internal pore structure and interact with the lipophilic humin deposits. However, the diffusion results clearly show that diffusivity is not significantly affected by the slight decrease in pore size that would accompany the surface deposition of humin.

## Conclusions

In conclusion, the deactivation profile of heterogeneous SBA-15-pr-SO_3_H catalysts for the alcoholysis of biobased FOL in ethanol is affected by the pore size of the catalysts. Deactivation is not due to leaching of the active organic moiety grafted on the surface but is mainly due to formation of humin deposits. The large-pore catalyst deactivates more rapidly compared to the small- and particularly medium-pore catalyst. This is due to the formation of larger amounts of humins, as demonstrated by EA. Humin formation affects also transport and adsorption properties. In particular, in larger-pore catalysts, molecules have greater access to the internal pore structure, as revealed by NMR relaxation; even though, humin deposition is not sufficient for blocking the pore in this case, the coverage on the internal surface results in a drop in reactivity.

## Experimental Section

### Materials and Methods

Pluronic P123 (average *M*_n_ ∼ 5800), diphenyl ether (99%), EL (98%), tetraethyl orthosilicate (98%), 3-MPTMS (95%), H_2_O_2_ aqueous solution (33% wt), and FOL (98%) were purchased from Merck Life Science (Milan, Italy). Concentrated hydrochloric acid and sulfuric acid were purchased from Carlo Erba (Milan, Italy). All solvents were dried by standard laboratory methods (distilled over proper drying agents or passed through anhydrous silica). GC analysis was performed on a Carlo Erba 6000 instrument, equipped with an FID and a Megadex 5 column (25 m × 0.25 mm) with the following temperature programs: 80 °C for 1 min, then 1 °C/min to 90 °C, and 25 °C/min to 190 °C; the retention times were 2.05 min for FOL and 6.10 min for EL; diphenyl ether was used as the external standard to quantify conversions and yields.

*T*_1_, *T*_2_, and diffusion measurements were performed on a Magritek benchtop 43 MHz SpinSolve using an inversion recovery, a CPMG, and a pulsed-field gradient stimulated echo (PGSTE) sequence, respectively.

FT-IR measurements were performed using a Bruker Vertex 70. EA was performed using a FLASH 2000 series CHNS/O analyzer (Thermo Fisher Scientific).

N_2_ adsorption–desorption isotherms were recorded on a Quantachrome Quadrasorb porosimeter. Samples were degassed at 150 °C for 18 h prior to recording N_2_ adsorption–desorption isotherms. BET surface areas were calculated over the relative pressure range of 0.02–0.2. Mesopore size properties were calculated using the Barrett, Joyner, and Halenda (BJH) method applied to the desorption branch of the isotherm.

XPS was performed on a Kratos Axis SUPRA X-ray photoelectron spectrometer fitted with a charge neutralizer and magnetic focusing lens using Al Kα monochromated radiation (1486.7 eV); spectral fitting was performed using CasaXPS version 2.3.19, with energy referencing to the C 1s peak of adventitious carbon at 284.6 eV. S 2p backgrounds were modeled using a quadratic function of cross-section (4535.29, −17.3355, 2704.68, −9) to account for the rising background from Si 2s photoelectron energy loss processes and subsequent Shirley-type function. Si 2p_3/2_ and 2p_1/2_ peaks were modeled using a line shape of LA (1.53,243), an energy separation of 1.15 eV, and an area ratio of 2:1.

Solid-state ^13^C NMR spectra were recorded using a Bruker 9.4 T (400 MHz ^1^H Larmor frequency) AVANCE III spectrometer equipped with a 4 mm HFX magic angle spinning (MAS) probe. Experiments were acquired at ambient temperature using an MAS frequency of 12 kHz. The sample was packed into a 4 mm o.d. zirconia rotor and sealed with a Kel-F rotor cap. The {^1^H-}^13^C cross-polarization (CP) NMR experiment employed spin-locking for 2 ms at ∼50 kHz for ^13^C, with corresponding ramped (70–100%) 73 kHz ^1^H spin-locking. 100 kHz SPINAL-64 heteronuclear ^1^H decoupling was used throughout signal acquisition.^64^ A Hahn-echo τ_r_–π–τ_r_ sequence of two rotor periods total duration was applied to circumvent receiver dead time. The ^13^C chemical shifts were referenced to tetramethylsilane externally.

*In situ* DRIFTS measurements were performed using a Bruker Vertex 70 FT-IR spectrometer equipped with a liquid N_2_-cooled mercury–cadmium–telluride detector. A catalyst sample (50–70 mg) was placed in the ceramic crucible of the *in situ* DRIFTS cell.

### **S1**/**S2**/**S3** (SBA-15 Support) Preparation

SBA-15 was synthesized using the procedure reported by Zhao *et al.*(65) Pluronic P123 (10 g) was dissolved in water (74.5 mL) and hydrochloric acid (2 M, 291.5 mL) and stirred at 35 °C. Tetraethyl orthosilicate (23.4 mL) was then added and left for 20 h under stirring. The resulting gel was aged under sealed conditions for 24 h (50 °C for small-pore-size SBA-15 synthesis, 80 °C for medium-pore-size SBA-15 synthesis, and 120 °C for large-pore-size SBA-15 synthesis) without stirring. The solid was filtered, washed with water (1000 mL), and dried at room temperature before calcination at 500 °C for 6 h in air (ramp rate of 1 °C/min). The pore size-controlled SBA-15 silicas were characterized by FT-IR, BET, BJH, and nitrogen sorption isotherms.

### **C1**/**C2**/**C3** (SBA-15-pr-SO_3_H Catalyst) Preparation

**C1**, **C2**, and **C3** were synthesized according to the procedure reported in the literature by a postgrafting approach:^66,67^ 1 g of SBA-15 (**S1**, **S2**, or **S3**) previously pretreated at 120 °C for 4 h under vacuum was suspended in dry toluene (25 mL) under mild stirring. 3-MPTMS was added (1.25 mL, 2.71 g, and 13.8 mmol), and the resulting opaque suspension was refluxed for 24 h to obtain the resulting propyl thiol group-modified SBA-15 (**C1**, **C2**, or **C3**). The solids were then filtered and washed with toluene (3 × 20 mL), acetone (3 × 20 mL), and *n*-hexane (3 × 20 mL). The materials were dried at 80 °C under vacuum. The thiol groups were converted into SO_3_H groups by treating the resulting SBA-15 with a H_2_O_2_ solution (20 mL, 30 wt %) under continuous stirring at 60 °C for 24 h. Finally, the solid was filtered, washed with water (3 × 20 mL), acidified under reflux conditions with 20 mL of a H_2_SO_4_ solution (10 wt %), filtered again, washed with water (3 × 20 mL), and then dried at 100 °C for 12 h under vacuum. The pore size-diversified SBA-15-pr-SO_3_H silicas were characterized by FT-IR, nitrogen sorption isotherms, EA, and XPS. Catalyst loadings and acid capacity (concentration of −SO_3_H groups) were determined by EA and titration, respectively. *General method for catalyst titration*: the concentration of −SO_3_H groups in SBA-15-pr-SO_3_H catalysts was determined by titration using a 0.01 M NaOH solution. In detail, 50 mg of the catalyst was added to 5 mL of deionized water and the suspension was stirred for 20 min. The suspension was then titrated with a NaOH solution using phenolphthalein as the indicator.

### Conversion of FOL into EL and Catalyst Recycling

General procedure for the synthesis of EL under batch conditions: **C1**, **C2**, or **C3** (100% w/w) was suspended in EtOH (4.5, 3.0, and 1.5 mL of the fresh catalyst, once-used catalyst, and twice-used catalyst, respectively) in a sealed Pyrex tube equipped with a poly(tetrafluoroethylene)-lined screw cap. FOL 0.3 M (132 mg, 88 mg, and 44 mg for each successive cycle) was added and the suspension was stirred at 120 °C for 16 h. Conversion *vs* time and yield *vs* time plots were obtained by cooling to room temperature and extracting 200 μL of the crude solution. This was then combined with 161 μL of the external standard solution (diphenyl ether, 0.414 M in ethyl acetate) and diluted to the mark with ethyl acetate before filtering for GC analysis.

For the catalyst recycling of the solid catalyst (**C1**, **C2**, or **C3**), the spent catalyst was collected from the reaction mixture *via* centrifugation. After collection of the supernatant liquid, the resulting brown solid was washed and centrifuged several times with fresh portions of EtOH (5 × 7.0 mL). The resulting SBA-15-pr-SO_3_H catalyst was recovered and dried (0.1 mbar, 40 °C, 16 h). A small amount was retained for analysis (EA, NMR, *etc.*), and the other part was directly used as the catalyst in the subsequent recycling experiment. Conversion of FOL over time (*t*) was calculated as [*M*_FOL(initial)_ – *M*_FOL(*t*)_]/*M*_FOL(initial)_. The yield of EL was calculated as *M*_EL(*t*)_/*M*_FOL(initial)_. The carbon content due to insoluble humin deposits can be estimated by the formula % C_(catalyst after *x* cycle)_ – % C_(fresh catalyst)_.

### Sample Preparation and Parameter Setup for NMR Relaxation and Diffusion Measurements

^1^H NMR relaxation measurements NMR experiments were performed on a Magritek SpinSolve 43 MHz benchtop NMR spectrometer as described in the Materials and Methods section.

Preparation of the solid samples for the NMR experiments was performed as follows: the catalyst (**C1**, **C2**, or **C3**) solid particles were soaked in the liquid for 24 h to ensure full saturation of the solid. The saturated solid samples were then transferred to 5 mm NMR tubes. To minimize errors due to evaporation of the liquid, a small amount of pure liquid was added dropwise onto a filter paper, which was placed under the cap of the NMR tube. The NMR tube was then placed into the magnet and left for approximately 20 min to achieve thermal equilibrium before measurements started.

For NMR measurements, a pulse length of 14 μs was used, with a pulse amplitude of **0** dB for the 90° pulse. The receiver gain was set to 40, and 16 384 points were acquired in the time domain with a dwell time of 20 μs.

Diffusion coefficients were determined using the PGSTE pulse sequence (Figure S1 and the typical data set in Figure S2). PGSTE experiments were performed by fixing the observation time Δ = 50 ms and using values of gradient pulse duration *δ* = 2–5 ms. The magnitude of the magnetic field gradient *g* was varied linearly with 16 spaced increments. In order to achieve full signal attenuation, maximum values of *g* of up to 163 mT m^–1^ were necessary. The diffusion coefficients *D* were calculated by fitting the Stejskal–Tanner equation to the experimental data.^68^

*T*_1_ was measured using the inversion recovery pulse sequence (Figure S3 and typical data set in Figure S4), with a repetition time of between 5 × *T*_1_, depending on the sample, and acquiring 16 time delay steps logarithmically spaced with a number of scans between 4 and 16, depending on the signal-to-noise ratio of the sample.

*T*_2_ was measured using the CPMG pulse sequence (Figure S5 and typical data set in Figure S6) using an echo time of 120 μs with 16 steps, using between 3 to 30 echoes per step and between 4 and 16 scans per step.

## Data Availability

The data that support the findings of this study are available within the article and the Supporting Information.

## Supporting Information Available

Catalyst and SBA-15 characterization and formulas (PDF)

## References

[ref1] Chaudhuri U. R. Petrochemicals. In Fundamentals of Petroleum and Petrochemical Engineering; CRC Press Taylor & Francis Group, 2010; pp 101–127.

[ref2] Abas N.; Kalair A.; Khan N. Review of Fossil Fuels and Future Energy Technologies. Futures 2015, 69, 31–49. 10.1016/j.futures.2015.03.003.

[ref3] Kusch-Brandt S. Urban Renewable Energy on the Upswing: A Spotlight on Renewable Energy in Cities in REN21’s “Renewables 2019 Global Status Report”. Resources 2019, 8, 139 10.3390/resources8030139.

[ref4] Perin G.; Jones P. R. Economic Feasibility and Long-Term Sustainability Criteria on the Path to Enable a Transition from Fossil Fuels to Biofuels. Curr. Opin. Biotechnol. 2019, 57, 175–182. 10.1016/j.copbio.2019.04.004. 31103911

[ref5] Brigham C. Biopolymers. In Green Chemistry; Elsevier, 2018; pp 753–770.

[ref6] Mishra M.; Sharma M.; Dubey R.; Kumari P.; Ranjan V.; Pandey J. Green Synthesis Interventions of Pharmaceutical Industries for Sustainable Development. Curr. Res. Green Sustainable Chem. 2021, 4, 100174 10.1016/j.crgsc.2021.100174.

[ref7] Kumar B.; Bhardwaj N.; Agrawal K.; Chaturvedi V.; Verma P. Current Perspective on Pretreatment Technologies Using Lignocellulosic Biomass: An Emerging Biorefinery Concept. Fuel Process. Technol. 2020, 199, 106244 10.1016/j.fuproc.2019.106244.

[ref8] Chang H.; Motagamwala A. H.; Huber G. W.; Dumesic J. A. Synthesis of Biomass-Derived Feedstocks for the Polymers and Fuels Industries from 5-(Hydroxymethyl)Furfural (HMF) and Acetone. Green Chem. 2019, 21, 5532–5540. 10.1039/c9gc01859j.

[ref9] Baraldi S.; Fantin G.; Di Carmine G.; Ragno D.; Brandolese A.; Massi A.; Bortolini O.; Marchetti N.; Giovannini P. P. Enzymatic Synthesis of Biobased Aliphatic–Aromatic Oligoesters Using 5,5′-Bis(Hydroxymethyl)Furoin as a Building Block. RSC Adv. 2019, 9, 29044–29050. 10.1039/c9ra06621g. 35528403 PMC9071804

[ref10] Lomba L.; Zuriaga E.; Giner B. Solvents Derived from Biomass and Their Potential as Green Solvents. Curr. Opin. Green Sustainable Chem. 2019, 18, 51–56. 10.1016/j.cogsc.2018.12.008.

[ref11] Mittal A.; Pilath H. M.; Johnson D. K. Direct Conversion of Biomass Carbohydrates to Platform Chemicals: 5-Hydroxymethylfurfural (HMF) and Furfural. Energy Fuels 2020, 34, 3284–3293. 10.1021/acs.energyfuels.9b04047.

[ref12] Rosatella A. A.; Simeonov S. P.; Frade R. F. M.; Afonso C. A. M. 5-Hydroxymethylfurfural (HMF) as a Building Block Platform: Biological Properties, Synthesis and Synthetic Applications. Green Chem. 2011, 13, 754–793. 10.1039/c0gc00401d.

[ref13] Dalvand K.; Rubin J.; Gunukula S.; Clayton Wheeler M.; Hunt G. Economics of Biofuels: Market Potential of Furfural and Its Derivatives. Biomass Bioenergy 2018, 115, 56–63. 10.1016/j.biombioe.2018.04.005.

[ref14] Shirotori M.; Nishimura S.; Ebitani K. One-Pot Synthesis of Furfural Derivatives from Pentoses Using Solid Acid and Base Catalysts. Catal. Sci. Technol. 2014, 4, 971–978. 10.1039/c3cy00980g.

[ref15] Danon B.; Marcotullio G.; de Jong W. Mechanistic and Kinetic Aspects of Pentose Dehydration towards Furfural in Aqueous Media Employing Homogeneous Catalysis. Green Chem. 2014, 16, 39–54. 10.1039/c3gc41351a.

[ref16] Mittal A.; Black S. K.; Vinzant T. B.; O’Brien M.; Tucker M. P.; Johnson D. K. Production of Furfural from Process-Relevant Biomass-Derived Pentoses in a Biphasic Reaction System. ACS Sustainable Chem. Eng. 2017, 5, 5694–5701. 10.1021/acssuschemeng.7b00215.

[ref17] Lange J.-P.; van der Heide E.; van Buijtenen J.; Price R. Furfural-A Promising Platform for Lignocellulosic Biofuels. ChemSusChem 2012, 5, 150–166. 10.1002/cssc.201100648. 22213717

[ref18] Mishra D. K.; Kumar S.; Shukla R. S. Furfuryl Alcohol—a Promising Platform Chemical. In Biomass, Biofuels, Biochemicals; Elsevier, 2020; pp 323–353.

[ref19] Rogers S. M.; Catlow C. R. A.; Chan-Thaw C. E.; Chutia A.; Jian N.; Palmer R. E.; Perdjon M.; Thetford A.; Dimitratos N.; Villa A.; Wells P. P. Tandem Site- and Size-Controlled Pd Nanoparticles for the Directed Hydrogenation of Furfural. ACS Catal. 2017, 7, 2266–2274. 10.1021/acscatal.6b03190.

[ref20] Villaverde M. M.; Bertero N. M.; Garetto T. F.; Marchi A. J. Selective Liquid-Phase Hydrogenation of Furfural to Furfuryl Alcohol over Cu-Based Catalysts. Catal. Today 2013, 213, 87–92. 10.1016/j.cattod.2013.02.031.

[ref21] Kumar A.; Srivastava R. Zirconium Phosphate Catalyzed Transformations of Biomass-Derived Furfural to Renewable Chemicals. ACS Sustainable Chem. Eng. 2020, 8, 9497–9506. 10.1021/acssuschemeng.0c02439.

[ref22] Hengne A. M.; Kamble S. B.; Rode C. v. Single Pot Conversion of Furfuryl Alcohol to Levulinic Esters and γ-Valerolactone in the Presence of Sulfonic Acid Functionalized ILs and Metal Catalysts. Green Chem. 2013, 15, 2540–2547. 10.1039/c3gc41098f.

[ref23] Song D.; An S.; Lu B.; Guo Y.; Leng J. Arylsulfonic Acid Functionalized Hollow Mesoporous Carbon Spheres for Efficient Conversion of Levulinic Acid or Furfuryl Alcohol to Ethyl Levulinate. Appl. Catal., B 2015, 179, 445–457. 10.1016/j.apcatb.2015.05.047.

[ref24] Mellmer M. A.; Gallo J. M. R.; Martin Alonso D.; Dumesic J. A. Selective Production of Levulinic Acid from Furfuryl Alcohol in THF Solvent Systems over H-ZSM-5. ACS Catal. 2015, 5, 3354–3359. 10.1021/acscatal.5b00274.

[ref25] Cheng X.; Feng Q.; Ma D.; Chen H.; Zeng X.; Xing F.; Teng J. Efficient Catalytic Production of Levulinic Acid over Hydrothermally Stable Propyl Sulfonic Acid Functionalized SBA-15 in γ-Valerolactone-Water System. J. Environ. Chem. Eng. 2021, 9, 105747 10.1016/j.jece.2021.105747.

[ref26] Pin J. M.; Guigo N.; Mija A.; Vincent L.; Sbirrazzuoli N.; van der Waal J. C.; de Jong E. Valorization of Biorefinery Side-Stream Products: Combination of Humins with Polyfurfuryl Alcohol for Composite Elaboration. ACS Sustainable Chem. Eng. 2014, 2, 2182–2190. 10.1021/sc5003769.

[ref27] Filiciotto L.; Balu A. M.; Romero A. A.; Angelici C.; van der Waal J. C.; Luque R. Reconstruction of Humins Formation Mechanism from Decomposition Products: A GC-MS Study Based on Catalytic Continuous Flow Depolymerizations. Mol. Catal. 2019, 479, 110564 10.1016/j.mcat.2019.110564.

[ref28] Shen H.; Shan H.; Liu L. Evolution Process and Controlled Synthesis of Humins with 5-Hydroxymethylfurfural (HMF) as Model Molecule. ChemSusChem 2020, 13, 513–519. 10.1002/cssc.201902799. 31746122

[ref29] van Zandvoort I.; Wang Y.; Rasrendra C. B.; van Eck E. R. H.; Bruijnincx P. C. A.; Heeres H. J.; Weckhuysen B. M. Formation, Molecular Structure, and Morphology of Humins in Biomass Conversion: Influence of Feedstock and Processing Conditions. ChemSusChem 2013, 6, 1745–1758. 10.1002/cssc.201300332. 23836679

[ref30] Xu Z.; Yang Y.; Yan P.; Xia Z.; Liu X.; Zhang Z. C. Mechanistic Understanding of Humin Formation in the Conversion of Glucose and Fructose to 5-Hydroxymethylfurfural in [BMIM]Cl Ionic Liquid. RSC Adv. 2020, 10, 34732–34737. 10.1039/d0ra05641c. 35514398 PMC9056862

[ref31] Sudarsanam P.; Zhong R.; van den Bosch S.; Coman S. M.; Parvulescu V. I.; Sels B. F. Functionalised Heterogeneous Catalysts for Sustainable Biomass Valorisation. Chem. Soc. Rev. 2018, 47, 8349–8402. 10.1039/c8cs00410b. 30226518

[ref32] Plutschack M. B.; Pieber B.; Gilmore K.; Seeberger P. H. The Hitchhiker’s Guide to Flow Chemistry. Chem. Rev. 2017, 117, 11796–11893. 10.1021/acs.chemrev.7b00183. 28570059

[ref33] De Risi C.; Bortolini O.; Brandolese A.; Di Carmine G.; Ragno D.; Massi A. Recent Advances in Continuous-Flow Organocatalysis for Process Intensification. React. Chem. Eng. 2020, 5, 1017–1052. 10.1039/d0re00076k.

[ref34] Chen C.; Volpe R.; Jiang X. A Molecular Investigation on Lignin Thermochemical Conversion and Carbonaceous Organics Deposition Induced Catalyst Deactivation. Appl. Energy 2021, 302, 117557 10.1016/j.apenergy.2021.117557.

[ref35] Argyle M.; Bartholomew C. Heterogeneous Catalyst Deactivation and Regeneration: A Review. Catalysts 2015, 5, 145–269. 10.3390/catal5010145.

[ref36] Kruk M.; Jaroniec M.; Ko C. H.; Ryoo R. Characterization of the Porous Structure of SBA-15. Chem. Mater. 2000, 12, 1961–1968. 10.1021/cm000164e.

[ref37] Zhao D.; Huo Q.; Feng J.; Chmelka B. F.; Stucky G. D. Correction to “Nonionic Triblock and Star Diblock Copolymer and Oligomeric Surfactant Syntheses of Highly Ordered, Hydrothermally Stable, Mesoporous Silica Structures”. J. Am. Chem. Soc. 2014, 136, 10546 10.1021/ja506344k.

[ref38] González Maldonado G. M.; Assary R. S.; Dumesic J.; Curtiss L. A. Experimental and Theoretical Studies of the Acid-Catalyzed Conversion of Furfuryl Alcohol to Levulinic Acid in Aqueous Solution. Energy Environ. Sci. 2012, 5, 6981–6989. 10.1039/c2ee03465d. PMC4097379 25035710

[ref39] Demma Carà P.; Ciriminna R.; Shiju N. R.; Rothenberg G.; Pagliaro M. Enhanced Heterogeneous Catalytic Conversion of Furfuryl Alcohol into Butyl Levulinate. ChemSusChem 2014, 7, 835–840. 10.1002/cssc.201301027. 24519990

[ref40] Zhao D.; Prinsen P.; Wang Y.; Ouyang W.; Delbecq F.; Len C.; Luque R. Continuous Flow Alcoholysis of Furfuryl Alcohol to Alkyl Levulinates Using Zeolites. ACS Sustainable Chem. Eng. 2018, 6, 6901–6909. 10.1021/acssuschemeng.8b00726.

[ref41] Johnson R. L.; Perras F. A.; Hanrahan M. P.; Mellmer M.; Garrison T. F.; Kobayashi T.; Dumesic J. A.; Pruski M.; Rossini A. J.; Shanks B. H. Condensed Phase Deactivation of Solid Brønsted Acids in the Dehydration of Fructose to Hydroxymethylfurfural. ACS Catal. 2019, 9, 11568–11578. 10.1021/acscatal.9b03455.

[ref42] Cantarutti C.; Dinu R.; Mija A. Biorefinery Byproducts and Epoxy Biorenewable Monomers: A Structural Elucidation of Humins and Triglycidyl Ether of Phloroglucinol Cross-Linking. Biomacromolecules 2020, 21, 517–533. 10.1021/acs.biomac.9b01248. 31675230

[ref43] Falco C.; Baccile N.; Titirici M.-M. Morphological and Structural Differences between Glucose, Cellulose and Lignocellulosic Biomass Derived Hydrothermal Carbons. Green Chem. 2011, 13, 3273–3281. 10.1039/c1gc15742f.

[ref44] Van Zandvoort I.; Koers E. J.; Weingarth M.; Bruijnincx P. C. A.; Baldus M.; Weckhuysen B. M. Structural Characterization of ^13^ C-Enriched Humins and Alkali-Treated ^13^ C Humins by 2D Solid-State NMR. Green Chem. 2015, 17, 4383–4392. 10.1039/c5gc00327j.

[ref45] Cantarutti C.; Dinu R.; Mija A. Biorefinery Byproducts and Epoxy Biorenewable Monomers: A Structural Elucidation of Humins and Triglycidyl Ether of Phloroglucinol Cross-Linking. Biomacromolecules 2020, 21, 517–533. 10.1021/acs.biomac.9b01248. 31675230

[ref46] Filippini G.; Longobardo F.; Forster L.; Criado A.; Di Carmine G.; Nasi L.; D’Agostino C.; Melchionna M.; Fornasiero P.; Prato M. Light-Driven, Heterogeneous Organocatalysts for C–C Bond Formation toward Valuable Perfluoroalkylated Intermediates. Sci. Adv. 2020, 6, abc9923 10.1126/sciadv.abc9923. PMC7673726 33177092

[ref47] Robinson N.; Robertson C.; Gladden L. F.; Jenkins S. J.; D’Agostino C. Direct Correlation between Adsorption Energetics and Nuclear Spin Relaxation in a Liquid-Saturated Catalyst Material. ChemPhysChem 2018, 19, 2472–2479. 10.1002/cphc.201800513. 29923663

[ref48] D’Agostino C.; Mitchell J.; Mantle M. D.; Gladden L. F. Interpretation of NMR Relaxation as a Tool for Characterising the Adsorption Strength of Liquids inside Porous Materials. Chem.—Eur. J. 2014, 20, 13009–13015. 10.1002/chem.201403139. 25146237 PMC4510707

[ref49] Di Carmine G.; Pesciaioli F.; Wang S.; Sinibaldi A.; Giorgianni G.; Parlett C. M. A.; Carlone A.; D’Agostino C. Insights into Substituent Effects of Benzaldehyde Derivatives in a Heterogenous Organocatalyzed Aldol Reaction. ChemCatChem 2022, 14, e202200405 10.1002/cctc.202200405.

[ref50] Di Carmine G.; Forster L.; Wang S.; Parlett C.; Carlone A.; D’Agostino C. NMR Relaxation Time Measurements of Solvent Effects in an Organocatalysed Asymmetric Aldol Reaction over Silica SBA-15 Supported Proline. React. Chem. Eng. 2022, 7, 269–274. 10.1039/d1re00471a.

[ref51] Di Carmine G.; Ragno D.; Massi A.; D’Agostino C. Oxidative Coupling of Aldehydes with Alcohol for the Synthesis of Esters Promoted by Polystyrene-Supported N-Heterocyclic Carbene: Unraveling the Solvent Effect on the Catalyst Behavior Using NMR Relaxation. Org. Lett. 2020, 22, 4927–4931. 10.1021/acs.orglett.0c01188. 32383888 PMC7341527

[ref52] D’Agostino C.; Stephens J. A.; Parkinson J. D.; Mantle M. D.; Gladden L. F.; Moggridge G. D. Prediction of the Mutual Diffusivity in Acetone–Chloroform Liquid Mixtures from the Tracer Diffusion Coefficients. Chem. Eng. Sci. 2013, 95, 43–47. 10.1016/j.ces.2013.03.033.

[ref53] D’Agostino C.; Mantle M. D.; Gladden L. F.; Moggridge G. D. Prediction of Mutual Diffusion Coefficients in Non-Ideal Mixtures from Pulsed Field Gradient NMR Data: Triethylamine–Water near Its Consolute Point. Chem. Eng. Sci. 2012, 74, 105–113. 10.1016/j.ces.2012.02.025.

[ref54] Abbott A. P.; D’Agostino C.; Davis S. J.; Gladden L. F.; Mantle M. D. Do Group 1 Metal Salts Form Deep Eutectic Solvents?. Phys. Chem. Chem. Phys. 2016, 18, 25528–25537. 10.1039/c6cp05880a. 27711611

[ref55] Haider M. H.; D’Agostino C.; Dummer N. F.; Mantle M. D.; Gladden L. F.; Knight D. W.; Willock D. J.; Morgan D. J.; Taylor S. H.; Hutchings G. J. The Effect of Grafting Zirconia and Ceria onto Alumina as a Support for Silicotungstic Acid for the Catalytic Dehydration of Glycerol to Acrolein. Chem.—Eur. J. 2014, 20, 1743–1752. 10.1002/chem.201302348. 24403184

[ref56] D’Agostino C.; Ryabenkova Y.; Miedziak P. J.; Taylor S. H.; Hutchings G. J.; Gladden L. F.; Mantle M. D. Deactivation Studies of a Carbon Supported AuPt Nanoparticulate Catalyst in the Liquid-Phase Aerobic Oxidation of 1,2-Propanediol. Catal. Sci. Technol. 2014, 4, 1313–1322. 10.1039/c4cy00027g.

[ref57] Isaacs M. A.; Robinson N.; Barbero B.; Durndell L. J.; Manayil J. C.; Parlett C. A.; D’Agostino C.; Wilson K.; Lee A. F. Unravelling Mass Transport in Hierarchically Porous Catalysts. J. Mater. Chem. A 2019, 7, 11814–11825. 10.1039/c9ta01867k.

[ref58] Forster L.; Lutecki M.; Fordsmand H.; Yu L.; D’Agostino C. Tailoring Morphology of Hierarchical Catalysts for Tuning Pore Diffusion Behaviour: A Rational Guideline Exploiting Bench-Top Pulsed-Field Gradient (PFG) Nuclear Magnetic Resonance (NMR). Mol. Syst. Des. Eng. 2020, 5, 1193–1204. 10.1039/d0me00036a.

[ref59] Jiao Y.; Forster L.; Xu S.; Chen H.; Han J.; Liu X.; Zhou Y.; Liu J.; Zhang J.; Yu J.; D’Agostino C.; Fan X. Creation of Al-Enriched Mesoporous ZSM-5 Nanoboxes with High Catalytic Activity: Converting Tetrahedral Extra-Framework Al into Framework Sites by Post Treatment. Angew. Chem., Int. Ed. 2020, 59, 19478–19486. 10.1002/anie.202002416. PMC7687177 32159268

[ref60] Barr M. R.; Forster L.; D’Agostino C.; Volpe R. Alkaline Pretreatment of Walnut Shells Increases Pore Surface Hydrophilicity of Derived Biochars. Appl. Surf. Sci. 2022, 571, 151253 10.1016/j.apsusc.2021.151253.

[ref61] D’Agostino C.; Mitchell J.; Mantle M. D.; Gladden L. F. Interpretation of NMR Relaxation as a Tool for Characterising the Adsorption Strength of Liquids inside Porous Materials. Chem.—Eur. J. 2014, 20, 13009–13015. 10.1002/chem.201403139. 25146237 PMC4510707

[ref62] Muhammad A.; Di Carmine G.; Forster L.; D’Agostino C. Solvent Effects in the Homogeneous Catalytic Reduction of Propionaldehyde with Aluminium Isopropoxide Catalyst: New Insights from PFG NMR and NMR Relaxation Studies. ChemPhysChem 2020, 21, 1101–1106. 10.1002/cphc.202000267. 32271976 PMC7317967

[ref63] Bräuer P.; Situmorang O.; Ng P. L.; D’Agostino C. Effect of Al Content on the Strength of Terminal Silanol Species in ZSM-5 Zeolite Catalysts: A Quantitative DRIFTS Study without the Use of Molar Extinction Coefficients. Phys. Chem. Chem. Phys. 2018, 20, 4250–4262. 10.1039/c7cp07826a. 29364297

[ref64] Fung B. M.; Khitrin A. K.; Ermolaev K. An Improved Broadband Decoupling Sequence for Liquid Crystals and Solids. J. Magn. Reson. 2000, 142, 97–101. 10.1006/jmre.1999.1896. 10617439

[ref65] Zhao D.; Feng J.; Huo Q.; Melosh N.; Fredrickson G. H.; Chmelka B. F.; Stucky G. D. Triblock Copolymer Syntheses of Mesoporous Silica with Periodic 50 to 300 Angstrom Pores. Science 1998, 279, 548–552. 10.1126/science.279.5350.548. 9438845

[ref66] Das D.; Lee J.-F.; Cheng S. Sulfonic Acid Functionalized Mesoporous MCM-41 Silica as a Convenient Catalyst for Bisphenol-A Synthesis. Chem. Commun. 2001, 21, 2178–2179. 10.1039/b107155f. 12240100

[ref67] Inagaki S.; Guan S.; Ohsuna T.; Terasaki O. An Ordered Mesoporous Organosilica Hybrid Material with a Crystal-like Wall Structure. Nature 2002, 416, 304–307. 10.1038/416304a. 11907572

[ref68] Stejskal E. O. Use of Spin Echoes in a Pulsed Magnetic-Field Gradient to Study Anisotropic, Restricted Diffusion and Flow. J. Chem. Phys. 1965, 43, 3597–3603. 10.1063/1.1696526.

