# Supplementary material for: Humin Formation on SBA-15-pr-SO3H Catalysts during the Alcoholysis of Furfuryl Alcohol to Ethyl Levulinate: Effect of Pore Size on Catalyst Stability, Transport, and Adsorption
Source: ACS Appl Mater Interfaces. 2023 May 15;15(20):24528–40. doi: 10.1021/acsami.3c04613 (PMC10214382; doi:10.1021/acsami.3c04613)
Supplement: Supplementary file 1 — am3c04613_si_001.pdf [file am3c04613_si_001.pdf]

Supporting Information

Humin Formation on SBA-15-pr-SO3H Catalysts during the Alcoholysis of Furfuryl Alcohol to Ethyl Levulinate: Effect of Pore Size on Catalyst Stability, Transport, and Adsorption

Graziano Di Carmine[a,*], Costanza Leonardi[a], Luke Forster[b], Min Hu[b], Daniel Lee[b], Christopher M. A. Parlett[b,c,d,] Olga Bortolini[e], Mark A. Isaacs[f,g], Alessandro Massi[a] and Carmine D'Agostino[b,h*]

[a]Department of Chemical, Pharmaceutical and Agricultural Sciences, University of Ferrara, Via L. Borsari, 46, 44121 Ferrara, Italy.

[b]Department of Chemical Engineering, University of Manchester, Oxford Road, M13 9PL, Manchester, United Kingdom.

[c]Diamond Light Source Harwell Science and Innovation Campus, OX11 0DE, Didcot, Oxfordshire, United Kingdom.

[d]Catalysis Hub, Research Complex at Harwell Rutherford Appleton Laboratory, OX11 0FA, Harwell, Oxfordshire, United Kingdom.

[e]Department of Environmental and Prevention Sciences, University of Ferrara, Via L. Borsari, 46, 44121 Ferrara, Italy.

[f]Department of Chemistry, University College London, London, WC1H 0AJ, United Kingdom.

[g]HarwellXPS, Research Complex at Harwell, RAL, Didcot, OX11 0FA, United Kingdom.

[h]Dipartimento di Ingegneria Civile, Chimica, Ambientale e dei Materiali (DICAM), Università di Bologna (UNIBO), via Terracini n. 28, 40131 Bologna, Italy.

*Corresponding authors

Dr Graziano Di Carmine

Email: dcrgzn@unife.it

Dr Carmine D'Agostino

Email: carmine.dagostino@manchester.ac.uk; carmine.dagostino@unibo.it

Table of Contents

NMR sample preparation and NMR measurements (*T_1*, *T_2* and diffusion) 3

XPS data for fresh and spent catalysts 7

Porosimetry analysis of fresh and spent catalysts 8

Evaluation of leaching after alcoholysis of furfuryl alcohol 8

Catalysts reactivity and selectivity 14

NMR sample preparation and NMR measurements (T_1, T_2 and diffusion)

Diffusion measurements

A standard PGSTE pulse sequence was used to determine imbibed *n*-octane diffusion coefficients (see **Figure S1** for the schematic representation of the pulse sequence).

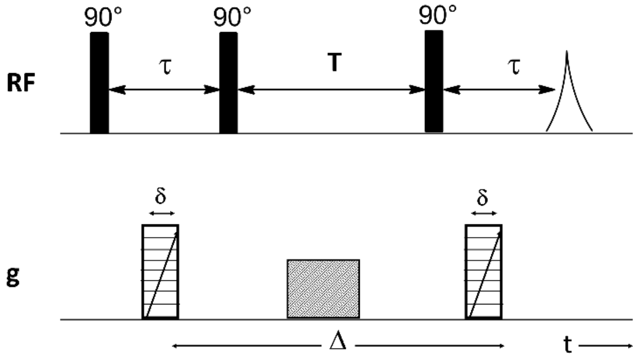

Figure S1. A schematic representation of PGSTE (Pulsed Gradient STimulated Echo) sequence.

Diffusion coefficients were obtained by fitting the data acquired with the following equation:

$$E(g)/E(0) = exp[-D\gamma^2\delta^2g^2(\Delta - \delta/3)] \qquad \textbf{(S1)}$$

In **Equation S1**, (g) and (0) are the NMR echo signal intensities in the presence and absence of the gradient, respectively. g is the gradient strength, δ is the gradient pulse length, γ is the gyromagnetic ratio of ^{1}H, Δ is the observation time, i.e., the time delay between the midpoints of the gradients and, finally, D is the diffusion coefficient. The term $[\gamma^2\delta^2g^2(\Delta - \delta/3)]$ is often referred to as the *b*-factor.

An example of diffusion experiment spectra recorded is shown in the **Figure S2** below.

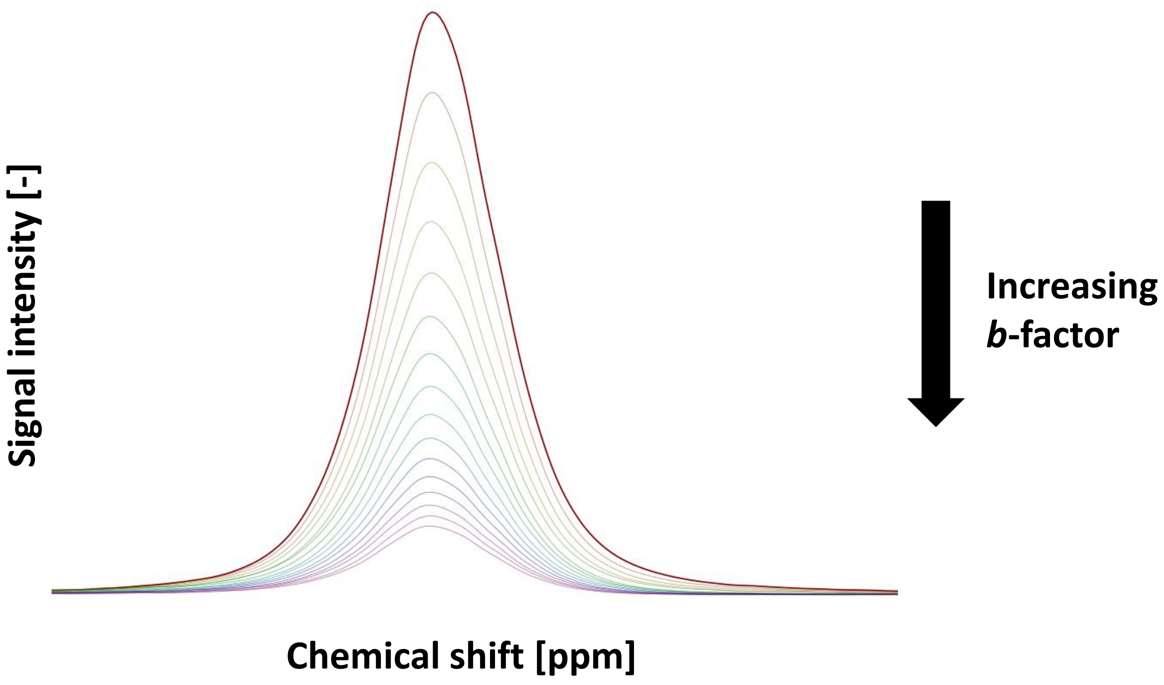

Figure S2. A typical set of diffusion spectra obtained from a PGSTE experiment.

***T_1* measurements**

A standard inversion recovery (IR) sequence was used to measure T_1 (see **Figure S3** for the schematic representation of the pulse sequence).

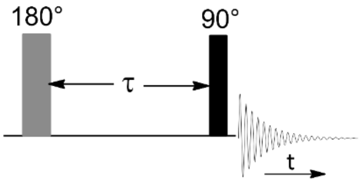

Figure S3. Inversion Recovery (IR) sequence.

The formula used to evaluate T_1 is the general **Equation S2** for the spin-lattice decay constant reported below. The FIDs were integrated and the values of the normalized signal intensity vs time were plotted to obtain T_1.

$$M_Z = M_0(1 - 2e^{-t/T_1}) \quad \textbf{(S2)}$$

An example of inversion recovery spectra recorded in this experiment is shown in the **Figure S4** below.

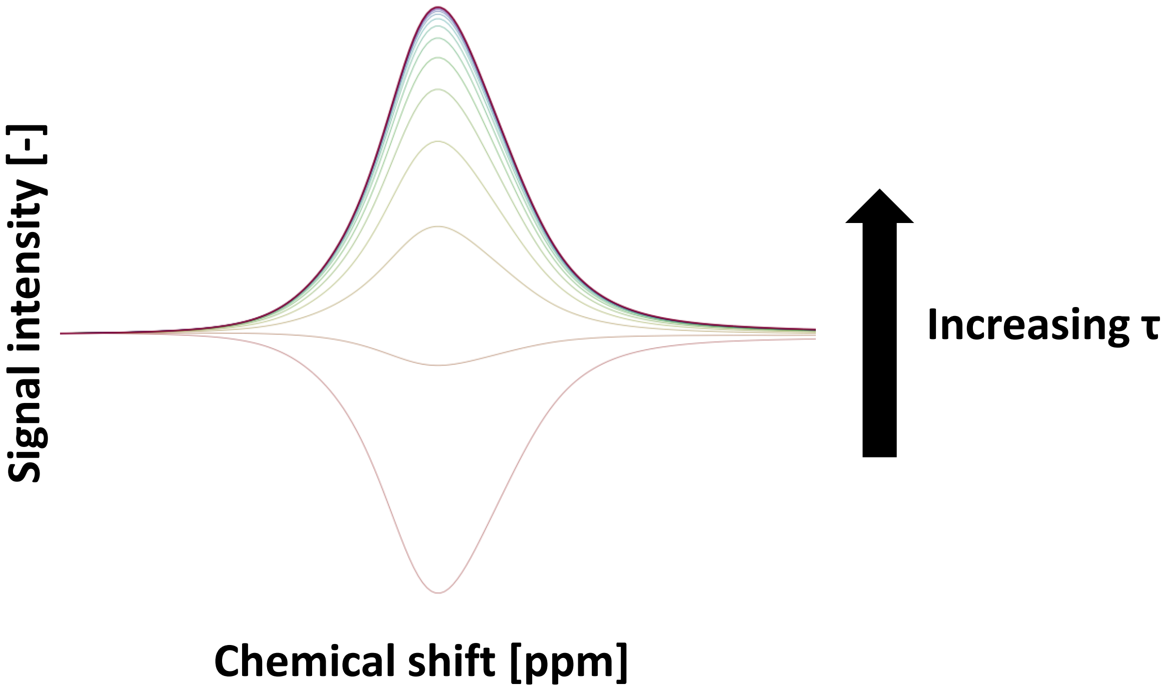

Figure S4. A typical set of spectra obtained from an inversion recovery experiment.

T_2 measurements

A Carr-Purcell-Meiboom-Gill (CPMG) pulse sequence was used to measure T_2 (see **Figure S5** for the schematic representation of the pulse sequence).

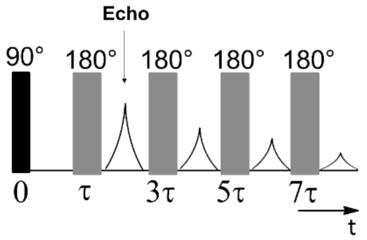

Figure S5. Carr-Purcell-Meiboom-Gill pulse sequence (CPMG).

The formula used to evaluate T_2 is the general **Equation S3** for the spin-spin decay constant reported below. The spectra were integrated and the values of the normalized signal intensity vs time were plotted to obtain the T_2.

$$M_{xy} = M_0 e^{-t/T_2} \tag{S3}$$

An example of CPMG set of spectra recorded in this experiment is shown in the **Figure S6** below.

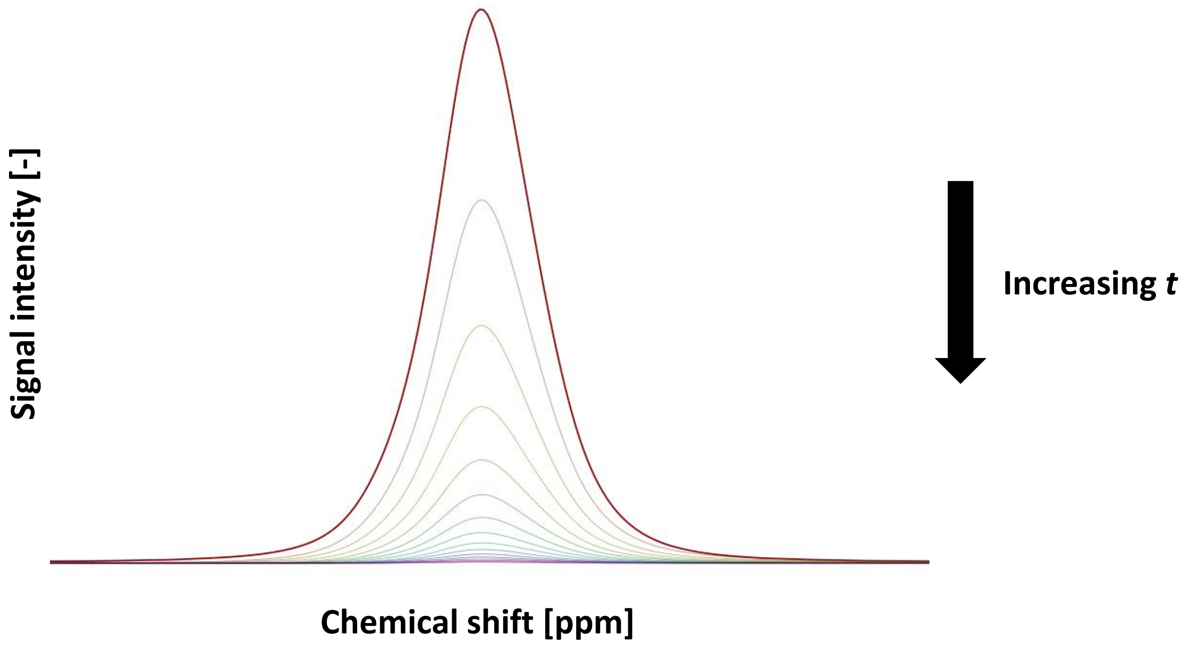

Figure S6. A typical set of spectra obtained from a CPMG experiment.

XPS data for fresh and spent catalysts

Table S1. Elemental quantification of fresh and spent catalysts. In the first column, the number in the brackets represent the number of cycles.

Sample Identifier	C 1s %	O 1s %	S 2p %	Si 2p %	Increase C 1S %	Increase C 1S %	Increase C 1S %
C3Fresh	3.34	65.59	0.47	30.60	0.00		
C3(I)	11.43	60.96	0.43	27.18	8.09		
C3(II)	15.97	58.26	0.35	25.41	12.64		
C3(III)	20.29	56.41	0.32	22.99	16.95		
C2Fresh	4.49	65.03	1.13	29.35		0.00	
C2(I)	8.75	63.09	0.54	27.62		4.26	
C2(II)	12.40	60.64	0.51	26.45		7.91	
C2(III)	14.94	59.95	0.45	24.67		10.44	
C1Fresh	4.13	65.46	0.69	29.72			0.00
C1(I)	13.24	59.96	0.72	26.08			9.11
C1(II)	31.75	49.16	0.44	18.65			27.62
C1(III)	27.62	51.76	0.53	20.09			23.49

Table S2. Quantification of C 1s. In the first column, the number in the brackets represent the number of cycles.

Sample Identifier	C-O %	C=O %	CC/CH %	O-C=O %	O 1s %	S 2p %	Si 2p %
C3Fresh	1.02	0.09	2.12	0.10	65.59	0.47	30.60
C3(I)	3.40	0.27	7.50	0.26	60.96	0.43	27.18
C3(II)	4.97	0.32	10.21	0.48	58.26	0.35	25.41
C3(III)	5.85	0.44	12.36	1.63	56.41	0.32	22.99
C2Fresh	1.97	0.12	2.40	0.00	65.03	1.13	29.35
C2(I)	3.20	0.21	5.34	0.00	63.09	0.54	27.62
C2(II)	3.86	0.25	8.00	0.29	60.64	0.51	26.45
C2(III)	5.33	0.32	8.89	0.39	59.95	0.45	24.67
C1Fresh	1.36	0.07	2.70	0.00	65.46	0.69	29.72
C1(I)	4.05	0.25	8.73	0.21	59.96	0.72	26.08
C1(II)	10.61	0.62	20.14	0.39	49.16	0.44	18.65
C1(III)	9.31	0.53	17.24	0.54	51.76	0.53	20.09

Porosimetry analysis of fresh and spent catalysts

Table S3. Structural properties of SBA-15-pr-SO_3H after recycling experiments.

SBA-15	Cycle	BET surface area (m^2/g)[a]	Average pore diameter (nm)[b]
**C1**	Fresh	752 ± 75	3.8 ± 0.4
	1	422 ± 42	3.6 ± 0.4
	2	309 ± 31	1.7 ± 0.2
	3	28.0 ± 2.8	2.2 ± 0.2
**C2**	Fresh	761 ± 76	4.8 ± 0.5
	1	600 ± 60	3.7 ± 0.4
	2	493 ± 49	3.7 ± 0.4
	3	371 ± 37	3.6 ± 0.4
**C3**	Fresh	686 ± 69	13.7 ± 1.4
	1	565 ± 56	12.8 ± 1.3
	2	475 ± 48	12.8 ± 1.3
	3	477 ± 48	12.0 ± 1.2

[a]calculated by BET analysis; [b]calculated by BJH analysis using the desorption branch.

Evaluation of leaching after alcoholysis of furfuryl alcohol

In order to evaluate the leaching of the catalytic moiety, the chemical formula of humin generated by acid catalyzed alcoholysis of furfuryl alcohol has been determined by elemental analysis. Samples were prepared as follows: **C1**, **C2** or **C3** (100 % w/w) was suspended in 5 mL of a solution of furfuryl alcohol in ethanol (0.3 M). The resulting suspension was sealed and stirred at 120 °C for 16 hours. Then, the resulting brown solid was filtered, washed with ethanol (5 × 5 mL) and dried under vacuum for 16 hours to remove unreacted FOL and reaction products. The solid was then stirred in a solution of NH_4HF_2 (50 mL, 1.0 M) for 16 hours. The

resulting suspension was filtered, washed several times with water (3 × 15 mL) and ethanol (3 × 15 mL) and finally dried under vacuum at 40 °C for 16 hours.

The elemental analysis provided the following results.

Table S4. Elemental analysis of humin formed in reaction with C1, C2 and C3 used for calculation of humin formula.

**Component name**	**% by weight (C1)**	**% by weight (C2)**	**% by weight (C3)**
Nitrogen	0	0	0
Carbon	71.55	72.31	73.40
Hydrogen	5.39	5.53	5.43
Sulphur	0	0	0

From these results the humin formula can be calculated and it is the same for each sample: $C_6H_5O_{1.5}$.

The structure of the functionalized SBA-15 is the -pr-SO_3H pendant, which is described below (**Scheme S1**). This is the only source of sulfur and carbon in the catalyst

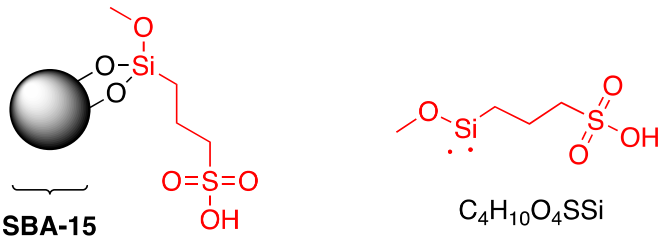

Scheme S1. Formula of the organic moiety anchored onto the silica SBA-15.

The derivation of the formula for the genuine calculation of catalyst loading is shown below. The percentage of carbon detected by EA is the sum of carbon that comes from humin, and the organic moiety anchored onto the SBA-15.

$$\%C_{tot} = \%C_{humin} + \%C_{organic\ moiety} \qquad \textbf{(S4)}$$

From rearrangement of **Equation S4**, a new expression is given (**Equation S5**).

$$\%C_{humin} = \%C_{tot} - \%C_{organic\ moiety} \quad \textbf{(S5)}$$

Therefore, the carbon percentage of the organic moiety can be determined by measuring the amount of sulfur. Based on the chemical formula of the organic moiety (**Scheme S1**) the amount (*mol*) of $C_{(organic\ moiety)}$ is four times that of $S_{(organic\ moiety)}$. Therefore, it follows that;

$$m_{C_{organic\ moiety}} = 4 * molS \quad \textbf{(S6)}$$

Thus, we can write that:

$$m_{C_{organic\ moiety}} = 4 * \frac{m_S * AW_C}{AW_S} \quad \textbf{(S7)}$$

where *AW* is the atomic weight. Thus:

$$\%C_{organic\ moiety} * \ m_{Tot} = 4 * \frac{\%S * m_{Tot} * AW_C}{AW_S} \quad \textbf{(S8)}$$

Through several simplifications:

$$\%C_{organic\ moiety} = 1.5 * \%S \quad \textbf{(S9)}$$

Substitution of the above **Equation S9** into **Equation S5** allows for the determination of $\%C_{humin}$:

$$\%C_{humin} = \%C_{tot} - 1.5 * \%S \quad \textbf{(S10)}$$

It is important to stress that the sulfur percentage measured from elemental analysis cannot be used to directly calculate the catalyst loading in the spent catalysts due to carbonaceous deposition that results in underestimation of the catalyst loading. In order to obtain the correct result, the mass amount from humin deposition must be removed. Thus, the following formula should be taken into account:

$$W_{humin} = W_{C\ humin} + W_{H\ humin} + W_{O\ humin} \quad \textbf{(S11)}$$

where W is weight (mass). This can be written as:

$$W_{humin} = AW_C * mol_{C\ humin} + AW_H * mol_{H\ humin} + AW_O * mol_{O\ humin} \quad \textbf{(S12)}$$

The assumed molecular formula of humin is $C_6H_5O_{1.5}$, as demonstrated above; thus, employing the stoichiometric coefficients it is possible to write the following formula:

$$mol_{C\ humin} = \frac{6}{5} mol_{H\ humin} = \frac{6}{1.5} mol_{O\ humin} \quad \textbf{(S13)}$$

Therefore, the number of moles of H and O can be expressed in terms of moles of carbon as shown in **Equation S14** and **Equation S15**.

$$mol_{H\ humin} = \frac{5}{6} mol_{C\ humin} \quad \textbf{(S14)}$$

$$mol_{O\ humin} = \frac{1.5}{6} mol_{C\ humin} \quad \textbf{(S15)}$$

Therefore, it follows that:

$$W_{tot-humin} = 1.403 * W_{C\ humin} \qquad \textbf{(S16)}$$

Thus:

$$\%W_{tot-humin} = 1.403 * \%C_{humin} \qquad \textbf{(S17)}$$

$\%C_{humin}$ of **Equation S10** can be replaced in **Equation S17** to obtain **Equation S18.**

$$\%W_{tot-humin} = 1.403 * (\%C_{tot} - 1.5 * \%S) \qquad \textbf{(S18)}$$

Sulfur, measured by EA of spent catalyst, is given as a percentage, taking into account the **weight of catalyst** ($W_{SBA\text{-}15}$ + $W_{organic\ moiety}$) and ($W_{tot\text{-}humin}$) the **weight of humin** deposited onto the surface. The total weight in spent catalyst (mass) is the sum of the masses of humin, SBA-15 and organic moiety:

$$W_{tot} = W_{tot-humin} + W_{SBA-15} + W_{organic\ moiety} \qquad \textbf{(S19)}$$

As we are interested in the *%S* for **weight of catalyst** ($W_{SBA\text{-}15}$ + $W_{organic\ moiety}$), here named *%S**.

$$\%S^* = \frac{W_S}{W_{SBA-15} + W_{organic\ moiety}} \qquad \textbf{(S20)}$$

We have that:

$$\%S^* = \frac{W_S}{W_{tot} - W_{tot-humin}} \qquad \textbf{(S21)}$$

Since:

$$\%S = \frac{W_S}{W_{tot}} \quad \Rightarrow \quad W_S = \%S * W_{tot} \tag{S22}$$

it follows that:

$$\%S^* = \frac{\%S}{1 - \frac{\%W_{tot-humin}}{100}} \tag{S23}$$

Replacing $\%W_{tot\text{-}humin}$ found in **Equation S18** in **Equation S23** the final equation is:

$$\%S^* = \frac{\%S}{(1 - \frac{1.403(\%C_{tot} - 1.5\%S)}{100})} \tag{S24}$$

Generally, catalyst loading can be calculated by EA as mmol g^{-1} from the following equation:

$$Cat\ Loading\ (mmol * \ g^{-1}) = \frac{\%S*}{AW_S} * 10 \tag{S25}$$

where $\%S*$ is equal to $\%S$ for fresh catalysts.

Catalysts reactivity and selectivity

The table below reports the yields of ethyl levulinate (EL) and conversions of furfuryl alcohol (FOL) at 4 hours of reaction; this reaction time has been selected to better compare the reactivity and selectivity among the catalysts. The formula employed for determining them are:

Conversion of FOL = $[M_{\mathrm{FOL(initial)}} - M_{\mathrm{FOL(t)}}]/M_{\mathrm{FOL(initial)}}$

Yield of EL = $M_{\mathrm{EL(t)}}/M_{\mathrm{FOL(initial)}}$

Carbon content due to insoluble humin deposits on used catalysts:

$\%C_{\mathrm{(catalyst\ after\ x\ cycle)}} - \%C_{\mathrm{(fresh\ catalyst)}}$

whereby *M* (molarity) has been measured by GC-FID as described in the experimental part.

Table S5. Yield, conversion and carbon content measured after each cycle for catalysts C1, C2 and C3.

Catalysts	Yield	Conversion	Carbon content
C1 (fresh)	70%	100%	-
C1 (I cycle)	56%	98%	5.5%
C1 (II cycle)	43%	79%	10.7%
C1 (III cycle)	-	-	14.7%
C2 (fresh)	70%	100%	-
C2 (I cycle)	65%	99%	3.4%
C2 (II cycle)	54%	90%	7.2%
C2 (III cycle)	-	-	10.5%
C3 (fresh)	59%	100%	-
C3 (I cycle)	33%	68%	6.7%
C3 (II cycle)	24%	61%	12.3%
C3 (III cycle)	-	-	16.2%

Even though full conversion of furfuryl alcohol is reached after 4 hours for all the catalysts, the yield of catalysts with larger pore size (C3) is lower than others. About recycling experiments, both conversion and yield drop after reusing, especially for larger pore size catalyst, as described in the text.

The figure below shows the kinetic profile (conversion (FOL) vs time and yield (EL) vs time) of reaction performed with **C1**, **C2** and **C3**.

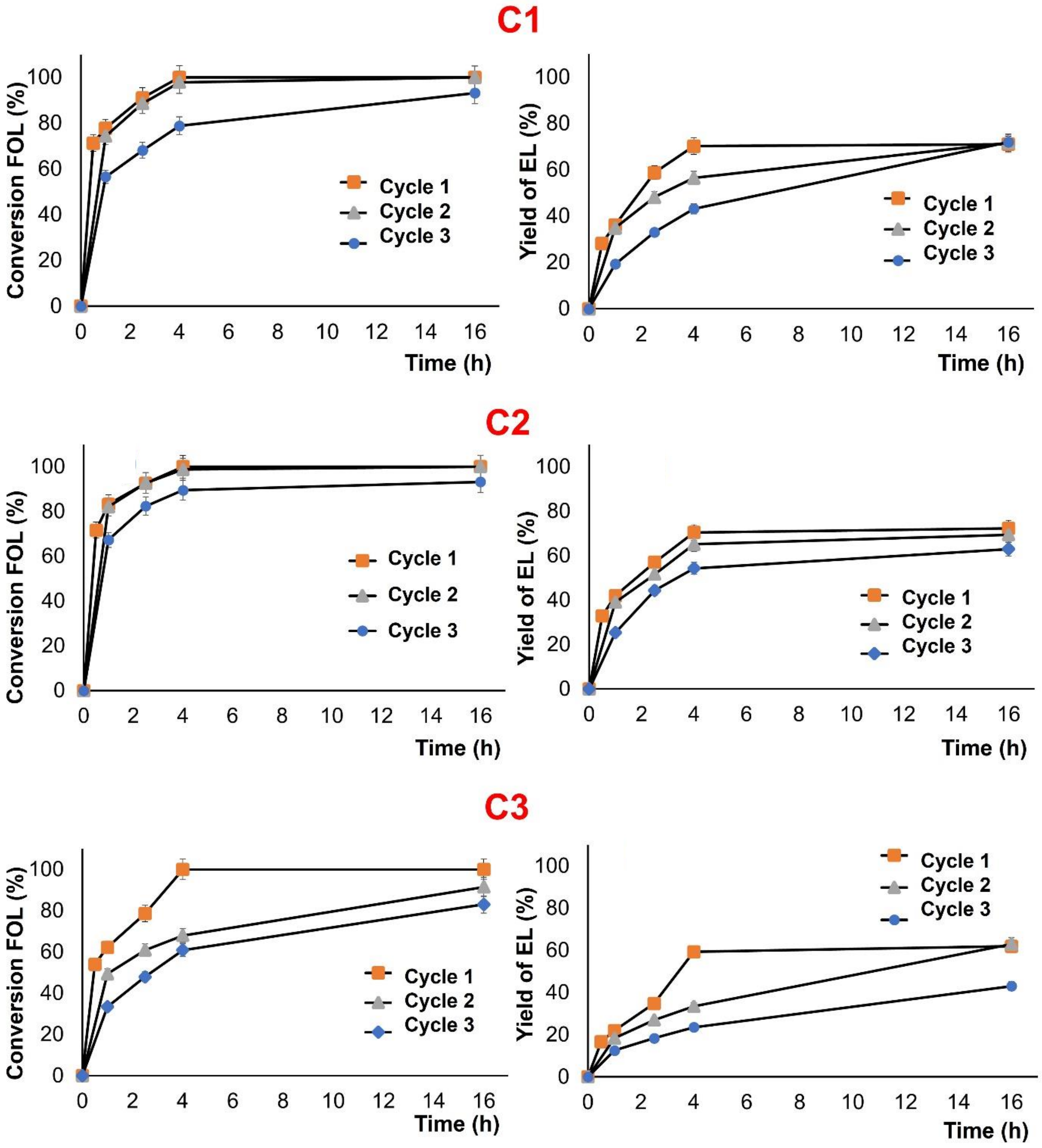

Figure S7. Reaction profiles and reusability tests for **C1** small, **C2** medium and **C3** large SBA-15-pr-SO_3H catalysts.
